# Control of replication stress and mitosis in colorectal cancer stem cells through the interplay of PARP1, MRE11 and RAD51

**DOI:** 10.1038/s41418-020-00733-4

**Published:** 2021-02-02

**Authors:** Gwenola Manic, Martina Musella, Francesca Corradi, Antonella Sistigu, Sara Vitale, Sara Soliman Abdel Rehim, Luca Mattiello, Eva Malacaria, Claudia Galassi, Michele Signore, Matteo Pallocca, Stefano Scalera, Frauke Goeman, Francesca De Nicola, Andrea Guarracino, Rosa Pennisi, Fabrizio Antonangeli, Francesca Sperati, Marta Baiocchi, Mauro Biffoni, Maurizio Fanciulli, Marcello Maugeri-Saccà, Annapaola Franchitto, Pietro Pichierri, Ruggero De Maria, Ilio Vitale

**Affiliations:** 1 IIGM - Italian Institute for Genomic Medicine, c/o IRCSS, Candiolo, Italy; 2 Candiolo Cancer Institute, FPO - IRCCS, Candiolo, Italy; 3 Dipartimento di Medicina e Chirurgia Traslazionale, Università Cattolica del Sacro Cuore, Rome, Italy; 4 Department of Biology, University of Rome “Tor Vergata”, Rome, Italy; 5 UOSD Immunology and Immunotherapy Unit, IRCCS Regina Elena National Cancer Institute, Rome, Italy; 6 Mechanisms, Biomarkers and Models Unit, Department of Environment and Health, Istituto Superiore di Sanità, Rome, Italy; 7 RPPA unit, Proteomics area, Core Facilities, Istituto Superiore di Sanità, Rome, Italy; 8 UOSD Biostatistics, Bioinformatics and Clinical Trial Center, IRCSS Regina Elena National Cancer Institute, Rome, Italy; 9 UOSD SAFU, Department of Research, Advanced Diagnostics, and Technological Innovation, Translational Research Area, IRCCS Regina Elena National Cancer Institute, Rome, Italy; 10 Oncogenomic and Epigenetic Unit, Department of Research, Advanced Diagnostics, and Technological Innovation, Translational Research Area, IRCCS Regina Elena National Cancer Institute, Rome, Italy; 11 Department of Experimental Oncology, European Institute of Oncology (IEO), Milano, Italy; 12 Department of Molecular Medicine, University “La Sapienza”, Laboratory affiliated to Istituto Pasteur Italia, Rome, Italy; 13 Institute of Molecular Biology and Pathology, National Research Council (CNR), Rome, Italy; 14 UOSD Biostatistics, Bioinformatics and Clinical Trial Center, San Gallicano Dermatological Institute IRCCS, Rome, Italy; 15 Department of Oncology and Molecular Medicine, Istituto Superiore di Sanità, Rome, Italy; 16 Division of Medical Oncology 2, IRCSS Regina Elena National Cancer Institute, Rome, Italy; 17 Fondazione Policlinico Universitario “A. Gemelli” - IRCCS, Rome, Italy

**Keywords:** Cancer stem cells, Preclinical research

## Abstract

Cancer stem cells (CSCs) are tumor subpopulations driving disease development, progression, relapse and therapy resistance, and their targeting ensures tumor eradication. CSCs display heterogeneous replication stress (RS), but the functionality/relevance of the RS response (RSR) centered on the ATR-CHK1 axis is debated. Here, we show that the RSR is efficient in primary CSCs from colorectal cancer (CRC-SCs), and describe unique roles for PARP1 and MRE11/RAD51. First, we demonstrated that PARP1 is upregulated in CRC-SCs resistant to several replication poisons and RSR inhibitors (RSRi). In these cells, PARP1 modulates replication fork speed resulting in low constitutive RS. Second, we showed that MRE11 and RAD51 cooperate in the genoprotection and mitosis execution of PARP1-upregulated CRC-SCs. These roles represent therapeutic vulnerabilities for CSCs. Indeed, PARP1i sensitized CRC-SCs to ATRi/CHK1i, inducing replication catastrophe, and prevented the development of resistance to CHK1i. Also, MRE11i + RAD51i selectively killed PARP1-upregulated CRC-SCs via mitotic catastrophe. These results provide the rationale for biomarker-driven clinical trials in CRC using distinct RSRi combinations.

## Introduction

Most human solid tumors, including colorectal cancer (CRC), are characterized by substantial interpatient and intratumor heterogeneity at genetic, epigenetic, transcriptional and/or phenotypic levels, representing a major obstacle to cancer treatment and patient survival [1–3]. A large body of evidence also indicates that tumors have a high cellular complexity often manifested with a hierarchical organization resembling that of the normal tissue [3, 4]. Indeed, marker-driven isolation, in vivo xenotransplantation, cell-ablation, lineage tracking, and single-cell genomics studies have revealed the presence, in CRC, of a mix of malignant cells with different degree of differentiation, maintained by a subpopulation of stem cell-like cells, known as cancer-stem cells (CSCs) [5–13]. In these studies, CSCs were detected in all steps of CRC development and shown to drive disease initiation, progression and dissemination.

The stemness-like properties of CSCs (i.e., self-renewal and multilineage differentiation potential), together with their unique therapeutic resistance, make these cells a major source of tumor heterogeneity and the culprit of treatment failure and tumor relapse [3, 14]. The specific targeting of CSCs is advocated to eradicate tumors, but other layers of complexity are emerging. It is now clear that cell types and states in tumors are dynamic features [15, 16]. Moreover, CSCs are reported to evolve both during disease progression and in response to therapy [17]. This explains the high heterogeneity in phenotypic markers, proliferation rate, tumorigenic/metastatic potential, karyotype, and treatment responsiveness observed across CSCs derived from CRC [18–24].

CSC heterogeneity represents a novel therapeutic challenge. Indeed, CSCs are endowed with a very robust DNA damage response (DDR), which drives resistance to genotoxic stress, but also constitutes a unique therapeutic vulnerability. However, such strong DDR in CSCs is reported to arise from multiple, non-mutually exclusive mechanisms, including enhanced DNA damage repair efficiency, DNA damage tolerance, antioxidant defenses as well as DDR signaling overactivation, apoptosis deregulation or senescence evasion (reviewed in [25]). Consequently, the targeting of one such unique CSC feature can be ineffective against some CSC subsets, and resistance can also emerge. So, it is urgent to reconstruct all the branches of the DDR in CSCs, and patient-derived experimental models capturing tumor genetic and phenotypic diversity can be suitably used to this aim [26].

One specialized branch of the DDR is dedicated to the management of replication stress (RS), so-called RS response (RSR) [27, 28]. RS is a form of genetic instability originating from the deregulation of the DNA replication process by endogenous or exogenous factors encompassing oncogenes, such as HRAS, MYC and CCNE1, or conventional anti-CRC chemotherapeutics, such as 5-FU, irinotecan and oxaliplatin [29–31]. These perturbations induce transient slowing or stalling of replication forks, resulting in the generation of long stretches of single-stranded DNA (ssDNA) at replication-fork junctions [28, 32]. The loading of ssDNA by the replication protein A (RPA) complex triggers the ATR-CHK1 axis of the RSR that arrests DNA replication and cell cycle progression through the activation of the intra-S checkpoint, also known as DNA replication checkpoint [28]. This limits further RS generation, replication factor exhaustion, and untimely mitotic entry [33], culminating either in the resolution/tolerance of DNA lesions or in the demise/senescence of cells too severely perturbed [28, 34, 35].

RS is frequently found in pre-cancerous lesions [36–38] and in established tumors [39, 40], but its levels appear heterogeneous in CSCs [21, 41]. Moreover, the mechanisms of RSR in CSCs have been poorly investigated so far. At this regard, the relevance of the RSR in CSCs is a matter of contentious [21, 41–43], and the actual functionality of the RSR in CSCs has been recently questioned [42]. Also, RSR abrogation variably affects CSC survival [27], as we previously showed in a panel of CRC patient-derived tumorspheres enriched for CSCs (CRC-SCs) treated with ATR or CHK1 inhibitors [21]. Finally, very few studies have specifically dealt with the development of resistance to RSR inhibitors in CSCs. Elucidating these issues could guide not only the design of specific prevention or rescue resistance therapies but also the identification of novel therapeutic vulnerabilities of CSCs.

Here, we demonstrated that the RSR in primary CRC-SCs is highly efficient and relies on the cooperation between the ATR-CHK1 axis and DDR players PARP1, RAD51 and/or MRE11. Building on these findings, we developed optimal, resistance-proof protocols targeting all CRC-SC subsets of our panel regardless of their genetic or ploidy profile.

## Results

### Impact of prolonged inhibition of the ATR-CHK1 axis on colorectal cancer stem cells (CRC-SCs)

**Fig. 1:**
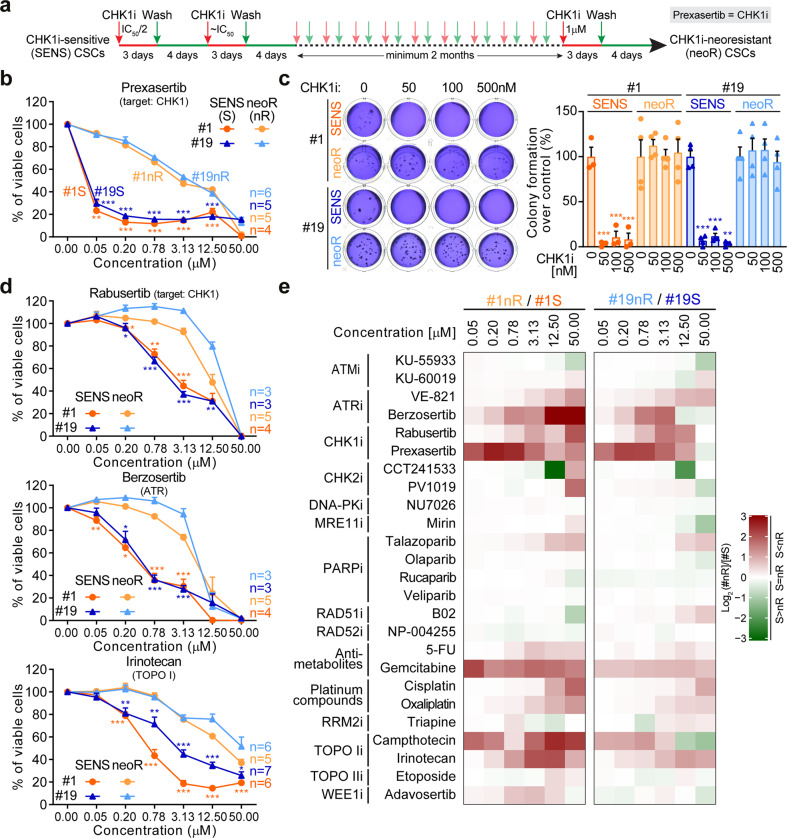
Effects of prolonged abrogation of the ATR-CHK1 axis in CRC-SCs. **a** Scheme of the experimental protocol used for the generation of neoR-CRC-SCs. For further details, see Materials and Methods. CHK1i refers to prexasertib, a preferential inhibitor of CHK1 and to a lesser extent CHK2. **b** Cell proliferation/viability of neoR-CRC-SCs vs. SENS-CRC-SCs left untreated or treated for 72 h with the indicated concentration of CHK1i, as assessed by CellTiter-Glo^®^ assay. Dose-response curves were calculated from the individual dose-response curves (each coming from an independent experiment) reported in Supplementary Fig. S1c. Results are expressed as means ± SEM, with number of independent experiments (*n*) for each group also reported. ^*^P < 0.05, ^**^P < 0.01, ^***^P < 0.001 compared to the corresponding neoR-CRC-SCs treated with the same dose (unpaired t-test with or without Welch’s correction). **c** Clonogenic survival of neoR-SENS-CRC-SCs vs. SENS-CRC-SCs left untreated or treated for 24 h with CHK1i, as indicated. Representative clonogenic assay images and quantitative data are reported. Results are expressed as means ± SEM and individual data points (*n* = 3 for #1SENS; *n* = 4 for #1neoR, #19SENS and #19neoR). ^*^P < 0.05, ^**^P < 0.01, ^***^P < 0.001 compared to untreated conditions for each CRC-SC (one-way ANOVA and Bonferroni or Dunnett T3 post-hoc test). **d** Dose-response curves of neoR-CRC-SCs vs. SENS-CRC-SCs left untreated or exposed for 72 h to ATR or CHK1 inhibitors or irinotecan as representative RS inducer. Proliferation/viability was assessed by CellTiter-Glo^®^ assay. Results are means ± SEM calculated from individual dose-response curves of Supplementary Fig. S1e. The *n* for each group is reported. ^*^P < 0.05, ^**^P < 0.01, ^***^P < 0.001 (unpaired t-test with or without Welch’s correction) compared to the corresponding neoR-CRC-SCs treated with the same dose. **e** Heatmap showing the differential sensitivity of neoR/SENS pairs to DDR inhibitors or activators. The values refer to log_2_ of the ratio of percentage of viable cells of neoR-CRC-SCs vs. SENS-CRC-SCs, calculated for each dose using data from dose-response curves of Fig. 1b, d, and Supplementary Figs. S1, S2. IC_50_ values for drugs differentially affecting neoR-CRC-SCs and SENS-CRC-SCs are in Supplementary Table S1, while all log2 values are in Supplementary Table S2. TOPO, topoisomerase. All significant *P* values are shown in Supplementary Table S4. Supplementary figures associated: Supplementary Figs. S1 and S2.

We previously showed that two-third of tumors contain CRC-SCs sensitive to the abrogation of the ATR-CHK1 axis of the RSR [21]. Among them, we can distinguish CRC-SCs hypersensitive or moderately sensitive to the inhibition of CHK1 by prexasertib (hereafter referred to as CHK1i) (Supplementary Fig. S1a). To investigate the RSR in CSCs, we first selected two functional subtypes of CRC-SCs: the KRAS wild-type #1 (CHK1i hypersensitive), and the KRAS mutated #19 (CHK1i moderately sensitive) [21]. These cells were relieved from their dependence on the ATR-CHK1 cascade by subjecting them to several consecutive rounds of treatments with CHK1i, starting from the IC_50_/2 value and gradually increasing the dose to 1 μM (Fig. 1a). Through this strategy, we favored the acquisition of resistance to CHK1i in both CRC-SCs, hereafter defined neo-resistant (neoR). CHK1i resistance was confirmed by a very low impact of CHK1i on survival (#1neoR IC_50_ = 2.9 μM; #19neoR IC_50_ = 4.1 μM), clonogenic/stemness potential and CD44v6^+^ stem-like fraction (Fig. 1b, c; Supplementary Fig. S1b, c). The concurrent administration of calcium channel blocker verapamil did not revert CHK1i resistance, ruling out a drug efflux phenomenon (Supplementary Fig. S1d). A drug-screening with 25 DDR targeting/triggering agents, including replication poisons commonly used to treat CRC in clinical practice, revealed that neoR-CRC-SCs were significantly more resistant than their sensitive counterparts (hereafter referred to as SENS) to the CHK1 inhibitor rabusertib and to the ATR inhibitors VE-821 and berzosertib (Fig. 1d, e; Supplementary Fig. S1e, f). On the contrary, no differences were found in CRC-SC response to inhibitors of the other DDR kinases ATM, CHK2 and DNA-PK, which were all less effective than ATR/CHK1 inhibitors (Fig. 1e; Supplementary Fig. S1f). Along with an insensitivity to ATR/CHK1 inhibition, neoR-CRC-SCs demonstrated a significant higher resistance than SENS-CRC-SCs to the clinically-relevant replication poisons irinotecan and, to a lesser extent, 5-FU and oxaliplatin (Fig. 1d, e; Supplementary Fig. S1e, f; Supplementary Tables S1, S2). This difference could be ascribed to the high sensitivity of SENS-CRC-SCs to irinotecan. Moreover, at least one neoR-CRC-SCs show increased resistance to most (but not all) RS-inducing agents, including gemcitabine, cisplatin, camptothecin and adavosertib (Fig. 1e; Supplementary Fig. S2), indicating an increased capability to resolve/tolerate severe RS.

Altogether, these findings demonstrate that prolonged impairment of the RSR by CHK1i results in increased resistance of CRC-SCs to clinically-relevant replication poisons and to inhibitors of the ATR-CHK1 cascade.

### Replication stress response is functional and efficient in CRC-SCs

**Fig. 2:**
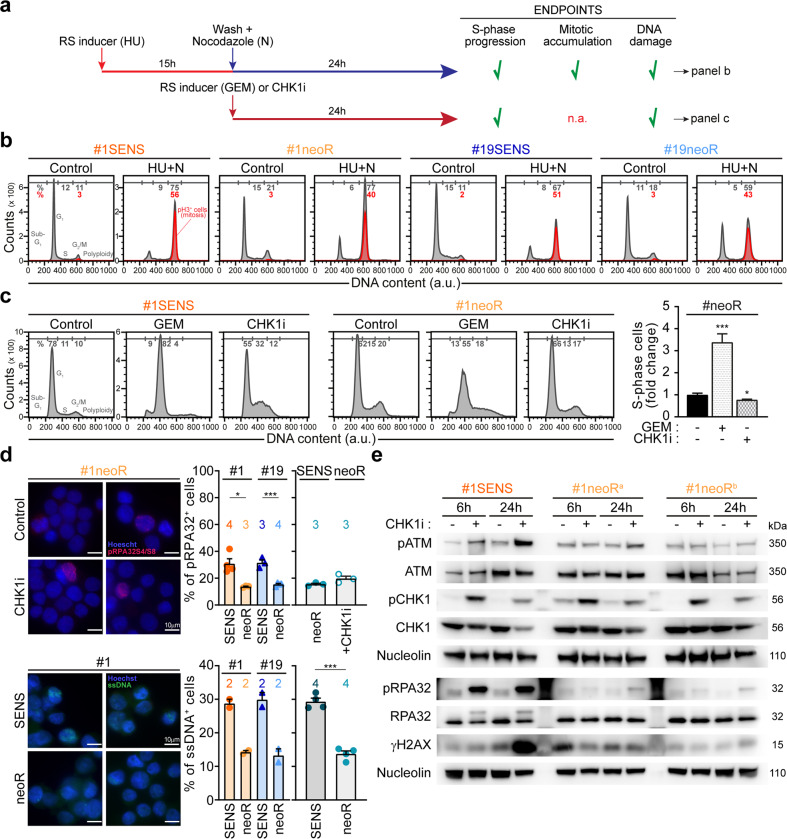
RSR is functional and efficient in CRC-SCs. **a**–**c** Evaluation of RSR proficiency in neoR/SENS-CRC-SCs subjected to exogenous replication stress (RS) as illustrated in the scheme in **a**. Specifically, cells were left untreated or either sequentially exposed to 1 mM hydroxyurea (HU) and, after drug washout, 1 µM nocodazole (N) (**a**, **b**; the so-called HU + N assay, see Materials and Methods), or treated for 24 h with 100 nM gemcitabine (GEM) or prexasertib (CHK1i) (**a**, **c**). Flow-cytometric cell cycle profiles upon staining with a DNA intercalant (DAPI) alone (**c**) or together with an anti-pH3 antibody (**b**) and quantitative data (**c**; means ± SEM from 6 independent experiments) are reported. In **b**, mitotic (pH3^+^) cells are in red. See also Supplementary Fig. S3a–c. ^*^P < 0.05, ^**^P < 0.01, ^***^P < 0.001 (one-way ANOVA and Dunnett T3 post-hoc test) compared to untreated conditions. **d** Immunofluorescence analysis in neoR-CRC-SCs and SENS-CRC-SCs left untreated or exposed for 24 h to 100 nM CHK1i and stained with an anti-pRPA32 antibody (top) or incubated with 10 μM IdU prior to anti-BrdU staining (bottom). Representative images and quantification of pRPA32^+^ cells and cells with parental ssDNA are reported. Results are expressed as means ± SEM and individual data points. Numbers refer to the number of independent experiments. ^*^P < 0.05, ^**^P < 0.01, ^***^P < 0.001 (unpaired t-test with or without Welch’s correction) as indicated. In the bottom part, statistical analysis was performed only in the histogram on the right. **e** Western-blot analysis in #1SENS-CRC-SCs and #1neoR-CRC-SCs treated or not with 100 nM CHK1i using the depicted antibodies (nucleolin as loading control). #1neoR^a^ and #1neoR^b^ correspond to cells collected after 12 and 17 weeks during CHK1i resistance generation. See also Materials and Methods and Supplementary Fig. S3d. All significant *P* values are shown in Supplementary Table S4. Supplementary figures associated: Supplementary Fig. S3.

The RSR status in CSCs is debated [27, 41–43]. Therefore, we exploited the newly-generated neoR/SENS pairs to investigate the RSR in CRC-SCs. We first explored RSR activity in CRC-SCs exposed to exogenous RS (Fig. 2a–c). Specifically, we performed a protocol of sequential administration of the RS inducer hydroxyurea (HU) and, after drug wash, of nocodazole (N), known as HU + N protocol [42, 44, 45] (Fig. 2a, b). In such assay, RSR-proficient cells can resolve severe RS, progressing in the cell cycle and arresting in metaphase, while cells with impaired RSR get stuck in the S-phase. We demonstrated that both neoR-CRC-SCs and SENS-CRC-SCs display an efficient RSR, as shown by low percentages of S-phase cells coupled with a huge increase in mitoses (Fig. 2b; Supplementary Fig. S3a) and absent DNA damage in most cells with replicated DNA (Supplementary Fig. S3b). Corroborating RSR proficiency, neoR-CRC-SCs activate the intra-S checkpoint, as revealed by the high accumulation of S-phase cells and DNA lesions after treatment with gemcitabine (Fig. 2c; Supplementary Fig. S3c).

We then explored the RSR in CRC-SCs not exposed to exogenous stress. We observed that untreated neoR-CRC-SCs display a significant decrease in constitutive RS compared to their SENS counterparts, manifested with a lower fraction of cells positive to pRPA32 or displaying parental ssDNA, two RS markers (Fig. 2d). Contrarily to their SENS counterparts [21], neoR-CRC-SCs treated with CHK1i did not experience severe RS, as shown by the low increase in the level of the RS markers pRPA32, pATM, and γH2AX (Fig. 2d, e; Supplementary Fig. S3d), and were not affected in their cell cycle progression (Fig. 2c; Supplementary Fig. S3c, e). Thus, in untreated conditions, low constitutive RS levels in neoR-CRC-SC make the ATR-CHK1 axis dispensable for survival. Accordingly, multiple agents boosting RS sensitized neoR-CRC-SCs to CHK1 and/or ATR inhibitors (Supplementary Fig. S3f–h).

Collectively, these observations demonstrate that the RSR is functional and efficient in CRC-SCs, but becomes dispensable for survival in CRC-SCs with low constitutive RS.

### PARP1 is upregulated and adjusts replication fork speed in resistant CRC-SCs

**Fig. 3:**
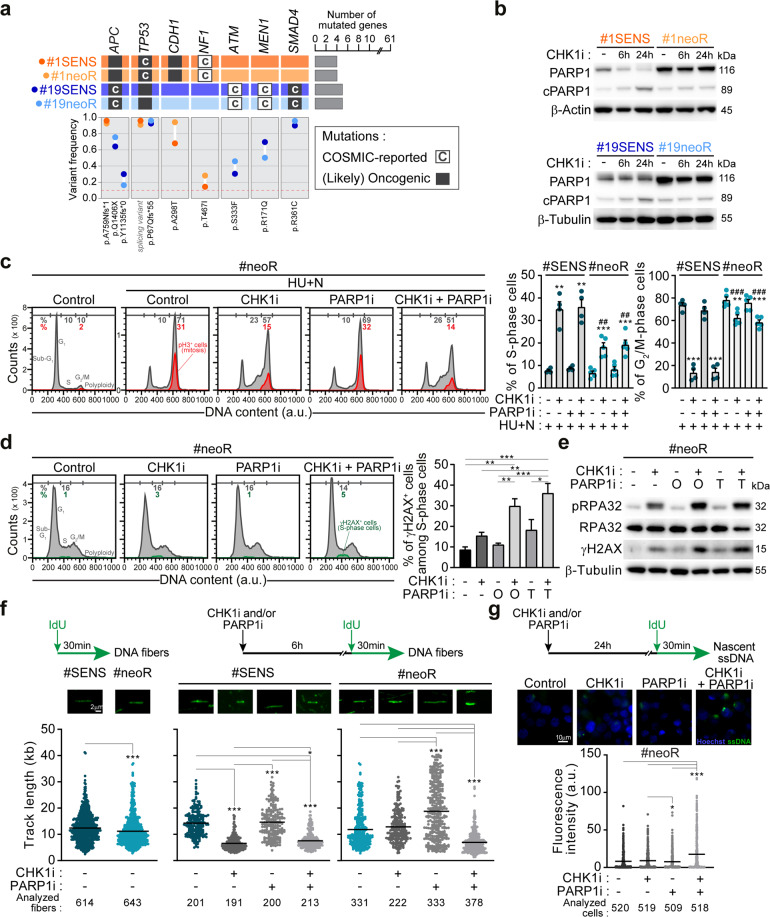
PARP1 is upregulated and modulates DNA replication speed in neoR-CRC-SCs. **a** Oncoprint of mutations for 61 DDR-related genes in neoR-CRC-SCs and SENS-CRC-SCs identified by deep sequencing. Only mutations included in COSMIC (“C”; http://cancer.sanger.ac.uk/cosmic) and/or annotated as (likely) oncogenic (dark gray squares) in the oncoKB database (https://oncokb.org/) are reported. Mutated gene number and allelic frequencies per CRC-SC are shown. Full gene list is in Supplementary Table S3. **b** Western-blot analysis in neoR-CRC-SCs and SENS-CRC-SCs left untreated or administrated for 6 h or 24 h with 100 nM prexasertib (CHK1i) and then stained with antibodies recognizing PARP1 and β-Actin or β-Tubulin (to ensure equal lane loading). cPARP1, cleaved PARP1. **c** Flow cytometry analysis in one representative CRC-SC pair (#1SENS/neoR) left untreated or sequentially treated with 1 mM hydroxyurea (HU) and, after drug washout, with 1 µM nocodazole (N) alone or together with CHK1i and/or PARP1i (talazoparib, TZ) (the HU + N assay, see Fig. 2a). Cell cycle profiles for #1neoR and quantification of S- and G_2_/M-phase cells for #1neoR and #1SENS are shown. Mitotic (pH3^+^) cells are in red. Results are expressed as means ± SEM and individual data points of 4 (#1SENS) or 5 (#1neoR) independent experiments. Cell cycle profiles of #1SENS are in Supplementary Fig. S4b. ^*^P < 0.05, ^**^P < 0.01, ^***^P < 0.001 (one-way ANOVA and Bonferroni or Dunnett T3 post-hoc test) compared to HU + N-treated conditions. ^#^*P* < 0.05, ^##^*P* < 0.01, ^###^*P* < 0.001 (unpaired t-test) compared to SENS-CRC-SCs. **d**, **e** Flow cytometry and immunoblot analysis of RS markers in neoR-CRC-SCs left untreated or treated with CHK1i and/or olaparib (O) or TZ (T) for 24 h (**d**) or 72 h (**e**) and stained with antibodies recognizing γH2AX, pRPA32, RPA32 and/or DAPI. Cell cycle profiles and quantification of γH2AX^+^ S-phase cells are reported in **d**. Results are means ± SEM of 5 independent experiments. ^*^P < 0.05, ^**^P < 0.01, ^***^P < 0.001 (one-way ANOVA and Bonferroni post-hoc test) as indicated (**d**). In **e**, β-Tubulin was used to ensure equal lane loading. See also Supplementary Fig. S4c. **f** DNA fiber assay in representative neoR/SENS-CRC-SCs (#19) left untreated or treated for 6 h with CHK1i and/or TZ and labeled with 250 µM IdU for the last 30 min. Representative DNA fiber images and dot-plots of IdU-labeled tract lengths for only untreated cells (left) or all conditions (right) are shown. Number of well-isolated DNA fibers per condition is reported. Data were pooled from four (left) and two (center and right) independent experiments (see Materials and Methods), and shown as box-plots with means and individual data points. ^*^P < 0.05, ^**^P < 0.01, ^***^P < 0.001, Mann–Whitney test for the comparison neoR-CRC-SCs vs. SENS-CRC-SCs (left), and Kruskal–Wallis ANOVA followed by Dunn’s post-hoc test for comparisons within neoR or SENS groups (center/right). **g** Immunofluorescence detection of nascent ssDNA in neoR-CRC-SCs left untreated or exposed to CHK1i and/or TZ for 6 h, labeled for the last 30 min of the treatment with 250 µM IdU, and finally stained with an anti-BrdU antibody. Representative images and quantification of cells displaying nascent strands (BrdU^+^) are shown. Data were pooled from two independent experiments and shown as box-plots with means and individual data points. Number of analyzed cells per condition is reported. ^*^P < 0.05, ^**^P < 0.01, ^***^P < 0.001 (Kruskal–Wallis ANOVA followed by Dunn’s post-hoc test). Dose range in **c–g**: 100–200 nM CHK1i, 5 µM OLA, 300–500 nM TZ; a.u. arbitrary units. All significant *P* values are shown in Supplementary Table S4. Supplementary figures associated: Supplementary Fig. S4.

To uncover unique mechanisms in the RSR of neoR-CRC-SCs, we performed targeted DNA sequencing analyses of a set of DDR-related genes in neoR/SENS pairs. Nonetheless, we detected no significant changes in the genetic background of neoR-CRC-SCs vs. SENS-CRC-SCs, and no commonly acquired/lost mutations in neoR-CRC-SCs (Fig. 3a; Supplementary Table S3). On the contrary, we revealed an increase in constitutive protein levels of PARP1 in untreated neoR-CRC-SCs compared to their SENS counterparts (Fig. 3b; Supplementary Fig. S4a).

Reportedly, PARP1 contributes to both DNA replication and DNA damage repair [46]. To explore the functional relevance of PARP1, we used the pharmacological inhibitors of PARP1/2 (hereafter referred to as PARP1i) olaparib and talazoparib assessing the response to RS. Through the HU + N assay, we observed correct S-phase progression, massive mitosis accumulation and low DNA damage persistence in both neoR-CRC-SCs and SENS-CRC-SCs challenged with HU and then coexposed to nocodazole and PARP1i (Fig. 3c; Supplementary Fig. S4b). On the contrary, CHK1i decreased the efficiency of neoR-/SENS-CRC-SCs to respond to severe RS by HU, an effect not modified by concurrent PARP1 inhibition (Fig. 3c; Supplementary Fig. S4b). This indicates that the ATR-CHK1 axis but not PARP1 is crucial in resolving severe RS induced by replication poisons. Intriguingly, RSR impairment by CHK1i was more pronounced in SENS-CRC-SCs than in neoR-CRC-SCs (Fig. 3c; Supplementary Fig. S4b), pointing to the emergence of additional mechanisms handling RS in neoR-CRC-SCs.

Next, we analyzed the role of PARP1 in DNA replication of CRC-SCs not exposed to exogenous stress. By flow cytometry, we revealed that, in neoR-CRC-SCs, PARP1i monotherapy did not significantly increase the levels of γH2AX in S-phase, a marker of RS fork breakage (Fig. 3d). On the contrary the concurrent administration of CHK1i and PARP1i boosted RS in neoR-CRC-SCs (Fig. 3d). Likewise, the CHK1i + PARP1i regimen augmented the levels of pRPA32 and γH2AX in immunofluorescence and western-blot (Fig. 3e; Supplementary Fig. S4c).

To explore DNA replication, we performed DNA fiber assay, allowing for visualizing/monitoring the dynamics of individual DNA replication forks [47]. In untreated conditions, we observed a slight but significant decrease of the replication track length in neoR-CRC-SCs vs. SENS-CRC-SCs, a sign of decelerated fork progression (by fork reversal [48]) or enhanced nuclease degradation at the replication fork (Fig. 3f). Intriguingly, CHK1i provoked nascent strand shortening in SENS-CRC-SCs but did not significantly modulate fork progression in neoR-CRC-SCs, which is in line with RS induction in the former. On the contrary, PARP1 inhibition led to a dramatic increase in DNA replication track length in neoR-CRC-SCs, a sign of accelerated DNA replication and increased fork progression speed, while did not affect track length of SENS-CRC-SCs (Fig. 3f). In line with the severe RS induction shown before, CHK1i + PARP1i diminished DNA replication track length also in neoR-CRC-SCs. These observations reveal a unique role of PARP1 in modulating fork speed progression in neoR-CRC-SCs.

To further explore replication fork integrity, we detected nascent ssDNA, a marker of DNA degradation at active replicating forks [49]. We observed low/absent nascent ssDNA in untreated and PARP1i-treated neoR-CRC-SCs, implying that PARP1 acts by modulating fork progression speed rather than nuclease degradation at replicating forks (Fig. 3g). Nascent ssDNA levels significantly increased only upon CHK1i + PARP1i (Fig. 3g), confirming that PARP1 inhibition boosts RS in neoR-CRC-SCs only when combined with ATRi/CHK1i.

Altogether, these findings demonstrate that PARP1 acts by adjusting replication fork speed in neoR-CRC-SCs.

### PARP1 inhibition reverts CSC resistance to ATR-CHK1 inhibitors inducing replication catastrophe

**Fig. 4:**
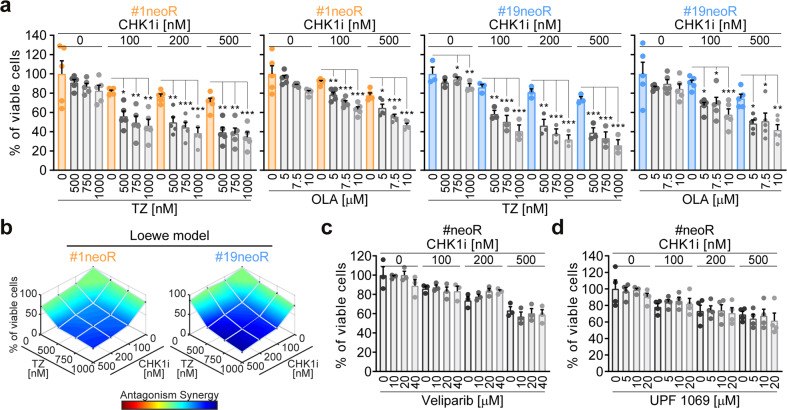
PARP1 inhibition sensitizes CRC-SCs to CHK1 inhibitors. **a**, **b** Cell viability (evaluated by CellTiter-Glo^®^ assay) of neoR-CRC-SCs exposed for 96 h with CHK1i alone or in combination with olaparib (OLA) or talazoparib (TZ), as indicated. Results are means ± SEM and individual data points of 5 (for OLA-treated #1neoR, TZ-treated #1neoR and OLA-treated #19neoR) or 3 (for TZ-treated #19neoR) independent experiments. ^*^P < 0.05, ^**^P < 0.01, ^***^P < 0.001 (one-way ANOVA and Bonferroni or Dunnett T3 post-hoc test) as reported. In **b**, synergism is calculated with the Combenefit software (see Materials and Methods). Similar results were found for doses of TZ not significantly decreasing neoR-CRC-SC survival when administered alone (100 and 250 nM; not shown). See also Supplementary Fig. S5a. **c**, **d** Cell viability (evaluated by CellTiter-Glo^®^ assay) of neoR-CRC-SCs left untreated or treated for 96 h with CHK1i alone or in combination with the indicated doses of PARP1i (veliparib, **c**) or PARP2i (UPF 1069, **d**) inhibitors, as indicated. Data from neoR-CRC-SCs were pooled. Results are means ± SEM and individual data points form 3 (**c**) and 4 (**d**) independent experiments. No statistical differences were observed (one-way ANOVA and Bonferroni or Dunnett T3 post-hoc test). All significant *P* values are shown in Supplementary Table S4. Supplementary figures associated: Supplementary Fig. S5.

We then assessed the impact of PARP1 upregulation on CSC survival. In line with drug-screening results, we confirmed that PARP1i alone did not affect neoR-CRC-SC survival (Fig. 4a). However, we showed that the inhibition of PARP1 significantly sensitizes neoR-CRC-SCs to CHK1i, exerting a synergistic effect (Fig. 4a, b; Supplementary Fig. S5a). Interestingly, CHK1i sensitization was observed for olaparib and talazoparib, which trap PARP1 on damaged DNA (PARP1 trappers [50, 51]), but not for the weak PARP1 trapper veliparib (Fig. 4c). Also, the specific PARP2i UPF 1069 did not increase CHK1i sensitivity (Fig. 4d), proving that such effect is PARP1-dependent. This evidence suggests that neoR-CRC-SC killing by CHK1i + PARP1i occurs through replication catastrophe [33].

**Fig. 5:**
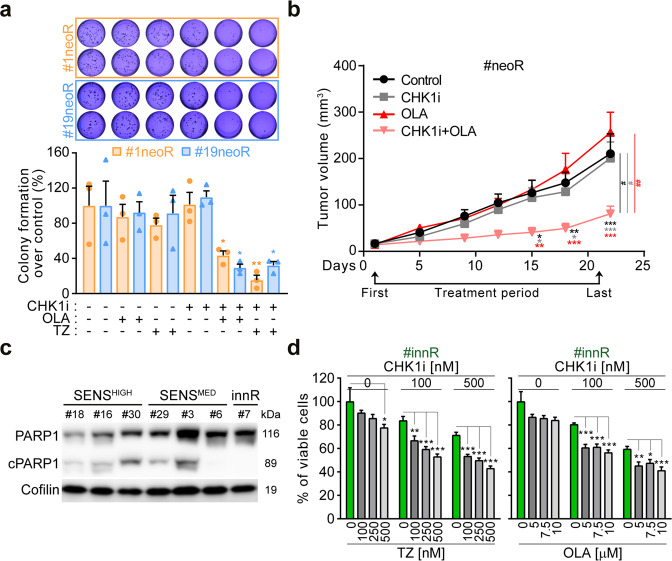
PARP1 inhibition boosts the in vivo and anticlonogenic effect of CHK1 inhibitors in CRC-SCs. **a** Clonogenic survival of neoR-CRC-SCs left untreated or pretreated for 72 h with 100 nM prexasertib (CHK1i) alone or together with 5 µM olaparib (OLA) or 300 nM talazoparib (TZ) and then cultivated in drug-free medium as indicated. Representative images and quantitative data are reported. Results are means ± SEM and individual data points from 3 independent experiments. ^*^P < 0.05, ^**^P < 0.01, ^***^P < 0.001 (one-way ANOVA and Bonferroni post-hoc test) compared to untreated conditions. See also Supplementary Fig. S5b. **b** In vivo growth of neoR-CRC-SCs (#19neoR) xenografted into immunodeficient NSG mice left untreated or treated with vehicles (Control), 5 mg/kg CHK1i, 50 mg/kg OLA or 5 mg/kg CHK1i + 50 mg/kg OLA as indicated (see Materials and Methods). NSG mice employed per group: Control- and OLA-groups: 7; CHK1i- and CHK1i + OLA-groups: 8). Tumor size curves are reported as means ± SEM. Arrows correspond to the first and last treatment, while line to the treatment period. ^*^P < 0.05, ^**^P < 0.01, ^***^P < 0.001 (two-way ANOVA and Bonferroni post-hoc test for multiple time-points) and ^#^*P* < 0.05, ^##^*P* < 0.01, ^###^*P* < 0.001 (Kruskal–Wallis ANOVA followed by Dunn’s post-hoc test for the last time point, vertical lines), as illustrated. **c** Western-blot analysis in CRC-SCs intrinsically resistant (innR), or moderately (SENS^MED^) or highly (SENS^HIGH^) sensitive to CHK1i exposed to 100 nM CHK1i for 24 h using antibodies against PARP1 and Cofilin (to ensure equal lane loading). cPARP1, cleaved PARP1. **d** Cell viability (evaluated by CellTiter-Glo^®^ assay) of innR-CRC-SCs treated with CHK1i alone or in the presence of OLA or TZ as illustrated. Data are reported as means ± SEM from 7 (on the left) and 8 (on the right) independent experiments. ^*^P < 0.05, ^**^P < 0.01, ^***^P < 0.001 (one-way ANOVA and Bonferroni or Dunnett T3 post-hoc test) as indicated. All significant *P* values are shown in Supplementary Table S4. Supplementary figures associated: Supplementary Fig. S5.

Importantly, the clonogenic survival of neoR-CRC-SCs was dramatically affected by CHK1i + PARP1i but not by CHK1i or PARP1i monotherapies (Fig. 5a; Supplementary Fig. S5b), confirming that CHK1i + PARP1i efficiently targets the CSC compartment. Consistently, CHK1i + olaparib significantly impaired the in vivo growth of neoR-CRC-SCs xenografted into the flank of immunodeficient NSG mice, while CHK1i/PARP1i monotherapies had no in vivo antitumor effect (Fig. 5b). Using our characterized CRC-SC panel (Supplementary Fig. S1a), we revealed that CRC-SC hypersensitive to CHK1i (SENS^HIGH^) display lower basal protein levels of PARP1 than CRC-SCs moderately sensitive to CHK1i (SENS^MED^) or innately resistant to CHK1i (innR) (Fig. 5c). Notably, PARP1i conferred sensitization to CHK1i also in innR-CRC-SCs, confirming the relevance of PARP1 in driving CRC-SC resistance to CHK1i (Fig. 5d).

These results demonstrate that PARP1 inhibition efficiently sensitizes former resistant CRC-SCs to CHK1i, both in vitro and in vivo, by inducing replication catastrophe.

### Targeting PARP1 prevents the acquisition of CSC resistance to ATR/CHK1 inhibitors

**Fig. 6:**
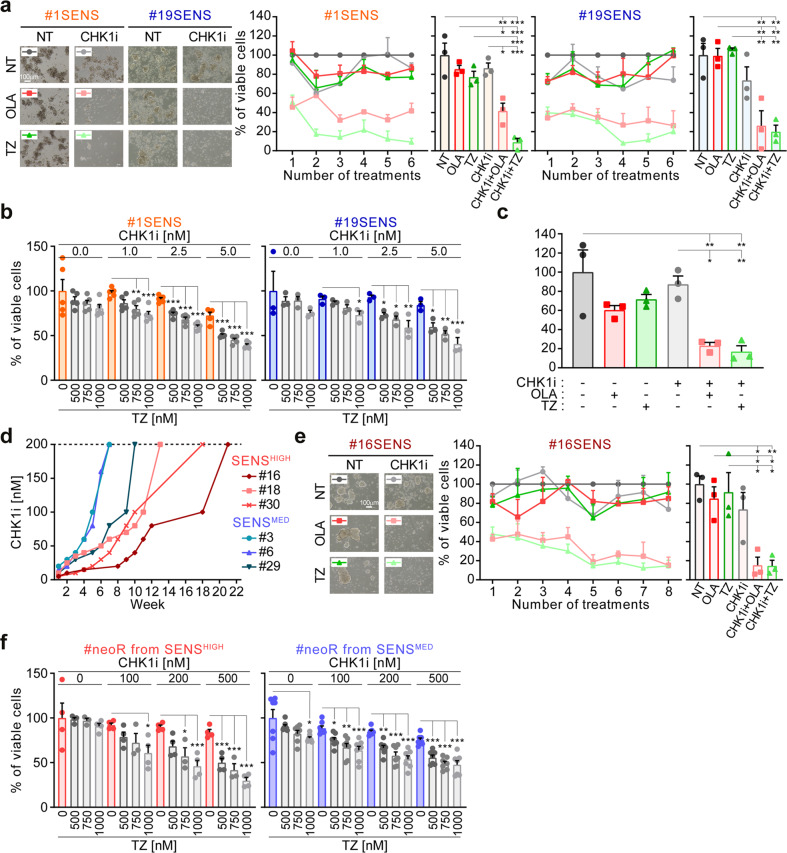
PARP1 inhibition prevents the generation of resistance to CHK1 inhibitors in CRC-SCs. **a** Cell viability of SENS-CRC-SCs left untreated or subjected to consecutive rounds of 72 h-treatment with prexasertib (CHK1i) alone or in combination with olaparib (OLA) or talazoparib (TZ) followed by ≥4 days of cultivation in drug-free medium, as described in Materials and Methods and Supplementary Fig. S6a. After each round of treatment, viable cells were counted upon Trypan Blue staining. Representative images and quantitative data of the percentage of viable cells at each treatment point or at the last time point are shown. Data are expressed as means ± SEM of 3 independent experiments. For the last time points, individual data points are also reported. The three individual experiments are in Supplementary Fig. S6b. ^*^P < 0.05, ^**^P < 0.01, ^***^P < 0.001 (one-way ANOVA and Bonferroni post-hoc test) compared to untreated conditions. Doses employed: CHK1i 1-30 nM; OLA 2-7 µM; TZ 100-500 nM. **b** Cell viability (assessed by CellTiter-Glo^®^ assay) of SENS-CRC-SCs left untreated or treated with sublethal doses of CHK1i and/or PARP1i for 96 h, as indicated. Results are reported as means ± SEM and individual data points from 5 (on the left) and 3 (on the right) independent experiments. ^*^P < 0.05, ^**^P < 0.01, ^***^P < 0.001 (one-way ANOVA and Bonferroni post-hoc test) as indicated. **c** Clonogenic survival of SENS-CRC-SCs left untreated or pre-treated for 72 h with 10 nM CHK1i, 5 µM OLA and/or 300 nM TZ. Quantitative data (#1SENS and #19SENS pooled) are shown. Results are reported as means ± SEM and individual data points from 3 independent experiments. ^*^P < 0.05, ^**^P < 0.01, ^***^P < 0.001 (one-way ANOVA and Bonferroni post-hoc test) as compared to the corresponding untreated CRC-SCs. Representative images are in Supplementary Fig. S6c. **d** Dynamics of the acquisition of resistance in SENS^HIGH^-CRC-SCs and SENS^MED^-CRC-SCs exposed to multiple rounds of CHK1i treatment using the protocol of Fig. 1a. CRC-SCs were considered resistant when they became insensitive to 200 nM CHK1i (dotted line: threshold). See also Supplementary Fig. S6e. **e** One representative SENS-CRC-SCs (#16SENS) was left untreated or subjected to consecutive rounds of treatments as in **a**. Representative images as well as quantitative data of the percentage of viable cells at each round of treatment(s) or at the last time point are shown. Data are expressed as means ± SEM of 3 independent experiments. Individual data points are also shown for the last time point. The three individual experiments are reported in Supplementary Fig. S6f. Data are analyzed as in **a**. **f** Cell viability of some neoR-CRC-SCs reported in panel **d** exposed for 96 h to CHK1i and/or TZ as indicated. Results are expressed as means ± SEM and individual data point from 4 independent experiments (on the left, with the exception of the 750 nM TZ-treated conditions in which is 3) and 7 independent experiments (on the right). Data on the right are pooled (#3+#6+#29neoR), while, on the left, results for #30neoR are reported. ^*^P < 0.05, ^**^P < 0.01, ^***^P < 0.001 (one-way ANOVA and Bonferroni or Dunnett T3 post-hoc test) as indicated. All significant *P* values are shown in Supplementary Table S4. Supplementary figures associated: Supplementary Fig. S6.

To make our results relevant for the clinic, we exploited the identified mechanism of resistance to CHK1i based on PARP1 upregulation as a means to prevent the acquisition of CHK1i resistance. To this aim, we added PARP1i during the neoR generation protocol (Supplementary Fig. S6a). Our rationale was to deplete the fraction of CRC-SCs with increased PARP1 levels once selected by CHK1i. Corroborating our hypothesis, PARP1i avoided the acquisition of resistance to CHK1i, leading (at least for talazoparib in most experiments) to the near-to-complete depletion of SENS-CRC-SCs (Fig. 6a; Supplementary Fig. S6b). Intriguingly, CHK1i + PARP1i decreased cell survival in SENS-CRC-SCs from the first cotreatment round. In line with this observation, the combination of sublethal doses of CHK1i and PARP1i efficiently killed SENS-CRC-SCs (Fig. 6b), also impairing clonogenicity (Fig. 6c; Supplementary Fig. S6c). We surmise that this sensitization is due to a further increase in RS by PARP1i, resulting in higher dependence on the ATR-CHK1 cascade. Accordingly, the administration of RS inducers sensitized SENS-CRC-SCs to sublethal doses of CHK1i (Supplementary Fig. S6d).

To further validate the role of PARP1 in resistance to ATRi/CHK1i, we generated neoR-CRC-SCs from 6 additional CRC-SCs with moderate/high sensitivity to CHK1i. We revealed that all SENS-CRC-SCs developed resistance to CHK1i, but SENS^MED^-CRC-SCs acquired resistance more rapidly than SENS^HIGH^-CRC-SCs (Fig. 6d; Supplementary Fig. S6e), presumably for their higher PARP1 levels. Taking advantage of these novel SENS/neoR pairs, we confirmed that PARP1i prevents the generation of resistance to CHK1i, sensitizing CRC-SCs to this agent (Fig. 6e, f; Supplementary Fig. S6f).

In conclusion, PARP1 inhibition not only sensitizes to, but also prevents the acquisition of resistance to CHK1i.

### Combined inhibition of MRE11 and RAD51 kills PARP1-upregulating CSCs by inducing mitotic catastrophe

**Fig. 7:**
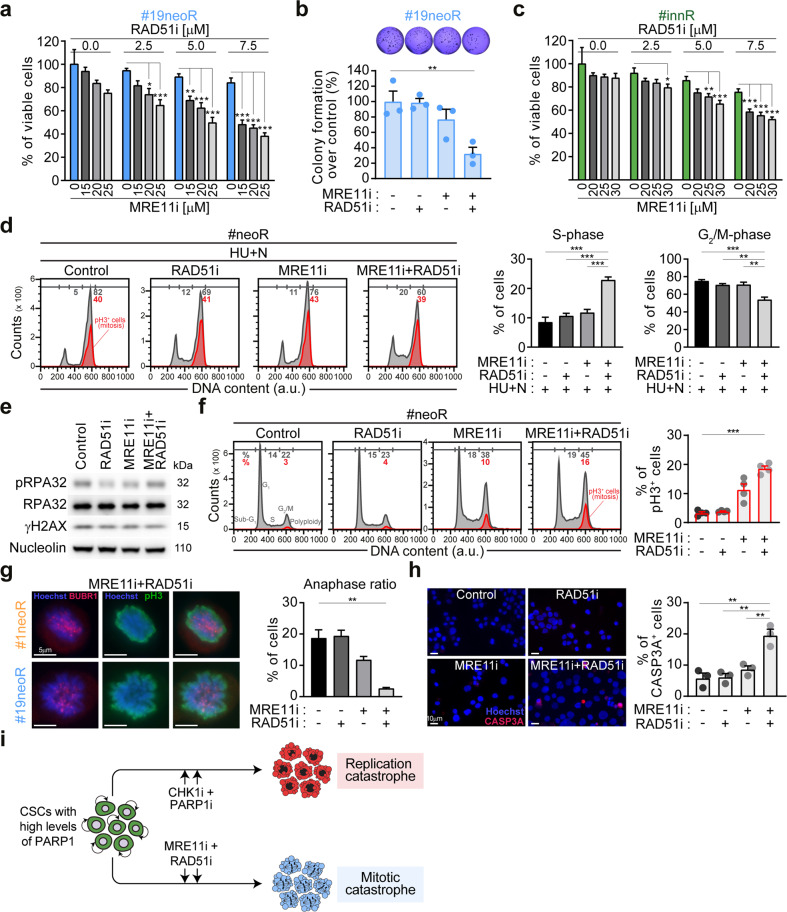
MRE11 and RAD51 cooperation contributes to RSR and mitosis and is essential in PARP1-upregulating CRC-SCs. **a**, **b** Cell viability (assessed by CellTiter-Glo^®^ assay) and clonogenic survival of neoR-CRC-SCs left untreated or exposed to inhibitors of MRE11 (mirin, MRE11i) and/or RAD51 (B02, RAD51i) for 72 h (**b**) or 96 h (**a**) as indicated. Data are reported as means ± SEM from 5 (**a**) or 3 (**b**) independent experiments. In **b**, representative images and individual data points are also shown (dose range: #19neoR: 15 µM MRE11i, 2.5 µM RAD51i). ^*^P < 0.05, ^**^P < 0.01, ^***^P < 0.001 (one-way ANOVA and Bonferroni post-hoc test), as indicated. See also Supplementary Fig. S7a–c. **c** Cell viability (assessed by CellTiter-Glo^®^ assay) of innR-CRC-SCs left untreated or exposed to inhibitors of MRE11 (mirin, MRE11i) and/or RAD51 (B02, RAD51i) or 96 h as indicated. Data are reported as means ± SEM from 9 independent experiments for all conditions with the exception of RAD51i 2.5 µM in which 5 independent experiments were performed. ^*^P < 0.05, ^**^P < 0.01, ^***^P < 0.001 (one-way ANOVA and Bonferroni or Dunnett T3 post-hoc test), as indicated. See also Supplementary Fig. S7d, e. **d–f** Flow cytometry and western-blot analyses of RSR functionality in representative neoR-CRC-SCs either treated with 1 mM hydroxyurea (HU) and, after drug washout, with 1 µM nocodazole (N) alone or together with MRE11i and/or RAD51i (the HU + N assay, see Fig. 2a) (**d**; #1neoR), or treated 24 h only with MRE11i and/or RAD51i (**e**; #1neoR; **f**; #19neoR). In **d** and **f**, cell cycle profiles (mitotic (pH3^+^) cells are in red) and quantification of S-phase or G_2_/M-phase or mitotic cells are represented. Results are reported as means ± SEM from 5 independent experiments (**d**) or as means ± SEM and individual data points from 4 independent experiments (**f**). ^*^P < 0.05, ^**^P < 0.01, ^***^P < 0.001 (one-way ANOVA and Bonferroni or Dunnett T3 post-hoc test) as depicted (**d**) or as compared to control condition (**f**). In **e**, antibodies recognizing γH2AX, pRPA32 and/or RPA32 and/or nucleolin (as loading control) were used. See also Supplementary Fig. S7f, g. **g** Immunofluorescence analysis in neoR-CRC-SCs treated with MRE11i and/or RAD51i for 24 h and then costained with antibodies against the spindle assembly checkpoint (SAC) activation marker BUBR1 (in red) and the mitotic marker pH3 (in green). Representative aberrant metaphases activating the SAC and the anaphase ratio (i.e., of the fraction of anaphases on 100 prophases+metaphases+anaphases, see Materials and Methods) are reported. Results are means ± SEM from 6 independent experiments. ^*^P < 0.05, ^**^P < 0.01, ^***^P < 0.001 (one-way ANOVA and Dunnett T3 post-hoc test) as compared to control condition. **h** Immunofluorescence analysis in neoR-CRC-SCs treated with MRE11i and/or RAD51i for 48 h and then stained with an anti-cleaved caspase-3 (CASP3A) antibody. Representative images and the quantification of CASP3A^+^ cells are shown. Results are expressed as means ± SEM and individual data points from 3 independent experiments. ^*^P < 0.05, ^**^P < 0.01, ^***^P < 0.001 (one-way ANOVA and Bonferroni post-hoc test) as indicated. **i** Proposed model. CRC-SCs resistant to therapeutically-relevant replication poisons or ATR-CHK1 inhibitors display low levels of RS and PARP1 upregulation and can be efficiently targeted by the combined inhibition of (i) CHK1 + PARP1, which provokes fork degradation and severe RS, resulting in lethal replication catastrophe, or (ii) RAD51 + MRE11, which deregulates RSR and mitosis, resulting in cell death via mitotic catastrophe. Dose range in **d–g**: #1neoR: 25 µM MRE11i, 7.5 µM RAD51i; #19neoR: 20 µM MRE11i, 5–7.5 µM RAD51i. All significant *P* values are shown in Supplementary Table S4. Supplementary figures associated: Supplementary Figs. S7 and S8.

To further extend the clinical potential of our results, we reasoned that the role of PARP1 in the RSR of neoR-CRC-SCs could make these cells vulnerable to the inhibition of other fork remodeling/stabilizing players, focusing on the druggable players MRE11 and RAD51 [52]. We observed that pharmacological inhibitors of MRE11 (by mirin) or RAD51 (by B02) were ineffective in depleting neoR-CRC-SCs (Fig. 7a; Supplementary Fig. S7a). On the contrary, the MRE11i + RAD51i regimen was effective against #19neoR-CRC-SCs and (to a lesser extent) #1neoR-CRC-SCs (Fig. 7a; Supplementary Fig. S7a, b). Moreover, MRE11i + RAD51i affected the clonogenic potential of neoR-CRC-SCs (Fig. 7b; Supplementary Fig. S7c) and the survival of innR-CRC-SCs and additional neoR-CRC-SCs (Fig. 7c; Supplementary Fig. S7d). This indicates effective targeting of the CSC compartment by MRE11i + RAD51i and a common dependence on the joint activities of MRE11 and RAD51 in CHK1i-resistant cells. Of note, SENS^MED^-CRC-SCs were more efficiently targeted by MRE11i + RAD51i than SENS^HIGH^-CRC-SCs (Supplementary Fig. S7e), possibly due to higher PARP1 levels. These observations indicate that the combined activities of MRE11 and RAD51 becomes particularly relevant for CRC-SCs in a PARP1-upregulated context.

To elucidate the mechanisms of cell death by MRE11i + RAD51i, we investigated the impact of MRE11 and RAD51 on the RSR of neoR-CRC-SCs. By HU + N assay, we found that MRE11i + RAD51i makes the RSR partially ineffective in neoR-CRC-SCs subjected to exogenous RS (Fig. 7d), suggesting that the cooperation between MRE11 and RAD51 can contribute to the RSR under severe RS. However, we observed that the inhibition of MRE11/RAD51, alone or in combination, did not markedly increase the level of RS in neoR-CRC-SCs (Fig. 7e; Supplementary Fig. S7f). Intriguingly, MRE11i + RAD51i did not affect S-phase progression, but induced a significant accumulation of metaphases (pH3^+^) (Fig. 7f; Supplementary Fig. S7g), an effect particularly evident in #19neoR and observable to a lesser extent with MRE11i. Accordingly, the concurrent inhibition of MRE11 and RAD51 impaired correct mitosis orchestration in neoR-CRC-SCs leading to accumulation of aberrant metaphases activating the spindle assembly checkpoint (SAC) [53] as shown by kinetochore localization of BUBR1 (BUBR1^+^pH3^+^ cells), along with an almost complete depletion of anaphases (Fig. 7g). Mitotic defects were accompanied by the induction of apoptosis as demonstrated by the increased incorporation of the vital dye propidium iodide (Supplementary Fig. S8) coupled to increased activation of caspase-3 (Fig. 7h) in neoR-CRC-SCs cotreated with MRE11i and RAD51i. These findings indicate that MRE11i + RAD51i kills neoR-CRC-SCs by mitotic catastrophe [54] with a pathway dependent on caspase activity.

In conclusion, we provided evidence of a novel functional interplay between MRE11 and RAD51 in the RSR and mitosis, which becomes essential, and hence exploitable therapeutically, in PARP1-upregulating CRC-SCs.

## Discussion

CSC depletion ensures tumor eradication, but the poor characterization of the mechanisms involved in genomic stability and drug resistance in CSCs together with their substantial heterogeneity constitute obstacles to the development of effective anti-CSC strategies. Here, we demonstrated that the RSR is functional in primary CRC-SCs, as we uncovered mechanistic insights in the RSR, which we successfully exploited for the design of long-term effective therapeutic protocols against CRC.

This study provides three major molecular insights with therapeutic implications. First, the actual functionality of the RSR in CSCs is a matter of debate. On the one hand, two studies on patient-derived ovarian cancer organoids and glioblastoma SCs reported that CSCs efficiently resolve RS [41, 43], in line with a large body of literature showing proficient ATR-CHK1 cascade activation by RS inducers in multiple CSC types [27]. On the other hand, a recent report on cancer cell lines and primary mammospheres showed an association between cancer stemness and RSR deficiencies [42]. Here, we demonstrated that primary CRC-SCs always display an efficient RSR. Our view to reconcile these results is that the RSR is specifically impaired in CSCs during tumor initiation, but a robust RSR is required in CSCs of established neoplasms endowing them with the capability to resist or tolerate RS. The evidence that the ATR-CHK1 cascade remains functional in RSR-impaired CSCs [42] suggests the existence of a hitherto unidentified inhibitory mechanism acting specifically during oncogenesis. Elucidating such mechanism and characterizing the RSR status of tumors at different disease stages are urgent needs for translating RSR-targeting strategies into the clinics.

Second, we provided evidence of a crucial role of PARP1 in CRC-SCs exhibiting low constitutive RS coupled to high resistance to (i) replication poisons including the standard CRC chemotherapeutic irinotecan, and (ii) ATR/CHK1 inhibitors including prexasertib (here dubbed CHK1i) and berzosertib which are under clinic investigation (https://clinicaltrials.gov). Specifically, we demonstrated that PARP1 is upregulated in these CSCs, encompassing both neoR-CRC-SCs and innR-CRC-SCs. We also showed that, although not directly involved in the response to severe exogenous RS, PARP1 contributes to basal genetic stability of resistant CRC-SCs by inducing a global slowdown of replication fork speed. Reportedly, fork deceleration arises from transient remodeling of stalled replication forks (so-called fork regression or reversal), which is modulated by PARP1 [55, 56]. Our hypothesis is that fork slowdown by PARP1 favors stalled fork stabilization in resistant CRC-SCs, providing extra-time to limit ssDNA accumulation and resolve endogenous RS. In support of this model, fork reversal was previously reported not only to be associated to active fork slowing upon exposure to replication poisons [57, 58], but also to mitigate endogenous RS in unperturbed S-phase [46]. Our results also suggest the existence of a coordination between PARP1 and the ATR-CHK1 signaling in setting constitutive RS levels in, and thus conferring therapeutic resistance to, CRC-SCs. We showed that, when administered alone, PARP1i accelerated DNA replication in neoR-CRC-SCs but did not significantly induce RS, resulting in a mild effect on their survival, in line with a recent study on primary ovarian cancer organoids [43]. On the contrary, coinhibition of PARP1 and CHK1 induced extensive fork degradation and severe RS in neoR-CRC-SCs, culminating in cell death due to lethal replication catastrophe [33] (Fig. 7i). The inhibition of CHK1, albeit not lethal, may be a contributing RS-inducing factor by promoting replication fork destabilization [59], which, when combined with fork deregulation by PARP1i, results in severe RS and CRC-SC death. It remains to be established whether PARP1 modulates replication speed in neoR-CRC-SCs by increasing regressed fork stability [55], preventing untimely fork resumption [55], regulating fork elongation [60] or acting directly on the DNA replication process [61].

Importantly, such role of PARP1 can be exploited therapeutically. Indeed, we showed that CHK1i + PARP1i decreased both the clonogenicity and in vivo growth of CRC-SCs resistant to clinically-relevant replication poisons, indicating effective targeting of the CSC compartment responsible for tumor re-growth and expansion [62]. That said, our results do not exclude that such regimen could also kill more differentiated cancer cells. Of note, by an original experimental approach, we demonstrated that the administration of PARP1i prevents the acquisition of resistance to CHK1i in CRC-SCs. This evidence is relevant as in our setting all SENS-CRC-SCs acquired resistance to CHK1i. It also supports the therapeutic use of PARP1i in combinatorial regimens with ATRi/CHK1i, as evaluated in ongoing clinical trials (https://clinicaltrials.gov).

The third major novelty of this study is the evidence of a functional link between MRE11 and RAD51 with crucial implication for genoprotection, mitosis and survival of PARP1-overexpressing CRC-SCs, including those resistant to irinotecan and ATRi/CHK1i. Specifically, we showed that MRE11 and RAD51 cooperate to ensure both an efficient response to severe RS and mitosis execution. The first action depends on the roles in the RSR of RAD51, which contributes to stressed replication fork stabilization, regression and restart in a homologous recombination (HR)-independent fashion [57, 63–66], and of MRE11, whose limited resection activity ensures efficient restart/repriming of stalled forks [67, 68]. The latter is possibly due to the role of MRE11 in the G_2_/M checkpoint and sister chromatid segregation [69–71]. As proof of the relevance of such coordinated function, the combined (but not single) inhibition of MRE11 and RAD51 effectively kills CRC-SCs displaying high PARP1 levels via a mitotic catastrophe process subsequent to RSR weakening and defective mitoses (Fig. 7i). Our hypothesis is that the coordinated activity of MRE11 and RAD51 becomes essential for CRC-SCs downstream of fork slowdown by PARP1, as it ensures reversed replication fork protection and timely restart, and, subsequently, accurate mitosis. Thus, beyond representing a mechanism of resistance and genoprotection [59, 63], fork reversal could also engender a unique vulnerability exploitable therapeutically to eradicate specific CRC-SC subsets. Future investigations will dissect the mechanisms of pathogenicity of deregulated fork remodeling in CRC-SCs and the link between RSR and cell division. Irrespective of these unknowns, our evidence is relevant therapeutically as MRE11i + RAD51i depletes CRC-SCs without the concurrent presence of endogenous/exogenous RS sources or exposure to ATR/CHK1 inhibitors.

In conclusion, in this study we contributed to shed light on the mechanisms of RSR in CSCs as we developed resistance-proof treatment options to eradicate CRC-SCs based on the association of specific markers related to the RSR (i.e., pRPA32 and PARP1). Importantly, these findings can be rapidly translated into biomarker-driven clinical trials, given that RSR markers as well as cancer stemness markers can easily be assessed experimentally in tumor specimens.

## Materials and methods

### Cell lines and culture conditions

Unless otherwise indicated, cell culture media was provided by Gibco-Thermo Scientific (Thermo Fisher Scientific, Waltham, MA), supplements and reagents by Sigma-Aldrich (Millipore-Sigma, Merck group, St. Louis, MO), and plasticware by Corning Life Sciences (Corning, NY) and Thermo Scientific. Patient samples were obtained in accordance with the standards of the institutional Ethics Committee on human experimentation (authorization no. CE5ISS09/282). CRC-SCs were isolated from these samples and cultured as detailed in [21, 72], and were all authenticated and validated for their in vivo stemness/self-renewal potential upon xenotransplanted into immunocompromised mice as reported in [73]. Cells were routinely confirmed to be Mycoplasma-free by PCR. CRC-SCs were routinely maintained in Dulbecco’s Modified Eagle Medium (DMEM)/F12-based (#52100047, #21700018) culture medium specific for CSCs containing 4 mg/mL bovine serum albumin (BSA; #A1312, US Biological, Salem, MA), 1X Penicillin-Streptomycin-Amphotericin B (PSF; #17-745E, Lonza, Basel, Switzerland), 0.13% NaHCO3 (#25080094, Thermo Scientific), 6 mM Hepes (#15630056, Thermo Scientific), 2mM L-glutamine (#25030024, Thermo Scientific), 0.1 mg/mL apotransferrin (#T2252), 0.4 units heparin sodium salt (#H3393), 1.1% glucose (#G8769), 25 µg/mL insulin (#91077 C), 6.3 ng/mL progesterone (#P8783), 9.7 µg/mL putrescine dihydrochloride (#P5780), and 5.2 ng/mL sodium selenite (#S5261) and supplemented with 20 ng/mL human epidermal growth factor (EGF; #AF-100-15, PeproTech, London, UK), 10 ng/mL human basic fibroblast growth factor (bFGF; #AF-100-18B, PeproTech), and 10 mM nicotinamide (#N0636). All CRC-SCs including neo-resistant (neoR) CRC-SCs (whose generation is described in the next session) were passaged once/twice a week at dilution 1:2 by mechanical dissociation, through a micropipette, and/or enzymatic dissociation, by incubating them for less than 5 min at 37 °C with TrypLE^™^ Select 10X (#A1217701, Thermo Scientific) and Accumax (#A7089) (1:1, V:V), and incubated in standard culture conditions in ultra-low attachment tissue culture flasks. In the experiments, cells were seeded as single cells onto the appropriate supports (6-, 24- or 96-well plates) 24 h before being subjected to treatment.

### Chemicals

The following compounds were provided by Selleck Chemicals (Houston, TX): 5-fluorouracil (#S1209), adavosertib (#S1525), B02 (#S8434), berzosertib (#S7102), campthotecin (#S1288), cisplatin (#S1166), etoposide (#S1225), gemcitabine (#S1714), GSK'872 (#S8465), hydroxyurea (#S1896), irinotecan (#S2217), KU-55933 (#S1092), KU-60019 (#S1570), mirin (#S8096), nocodazole (#S2775), NU7026 (#S2893), olaparib (#S1060), oxaliplatin (#S1224), prexasertib (#S7178), Q-VD-Oph (#S7311), rabusertib (#S2626), rucaparib (#S1098), talazoparib (#S7048), triapine (#S7470), UPF 1069 (#S8038) VE-821 (#S8007), and veliparib (#S1004). Verapamil (#V4629) were provided by Sigma-Aldrich, CCT241533 (#HY-14715B) by MedChem Express (Monmouth Junction, NJ), NP-004255 (#PC-61922) by ProbeChem (Shanghai, China), and PV1019 (#220488) by Calbiochem-Merck-Millipore (Billerica, MA). The appropriate amount of DMSO (#5879) was employed for negative control conditions.

### Protocol for the generation of neo-resistant (neoR) CRC-SCs

To stimulate the development of resistance to prexasertib (CHK1i), we selected multiple CRC-SCs from our panel previously characterized as sensitive to CHK1i, namely the hypersensitive (or highly sensitive, SENS^HIGH^) CRC-SCs #1 (Figs. 1–7), #16, #18, and #30 (Figs. 5c and 6d, e) and the moderately sensitive (or medium-sensitive, SENS^MED^) CRC-SCs #19 (Figs. 1–7), #3, #6, and #29 (Figs. 5c and 6d). For further details on the genetic and cytogenetic background of CRC-SCs of our panel and for the classification of CHK1i sensitivity and the IC_50_ values of CHK1i, see [21] and Supplementary Fig. S1a. In these cells, CHK1i resistance was induced as follow. CRC-SCs were dissociated as single cells and then cultured for 24 h in T75-flask and treated with CHK1i, starting from their IC_50_/2 value concentration (i.e., 5 nM for #1 and 29 nM for #19) to promote resistance to CHK1i. After 72 h, cells were washed with PBS and cultured in fresh medium for at least 72 h. Several rounds of CHK1i administration were performed according to the response of cells to each treatment, using increasing concentrations of CHK1i until reaching the dose of 1 μM. In experiments aimed at analyzing the effect of PARP1i in preventing the acquisition of CHK1i resistance (Fig. 6), SENS-CRC-SCs were subjected to the same protocol of CHK1i administration also adding PARP1i at varying doses according to their relative sensitivity (dose range for olaparib: 2–7 µM (#1SENS and #19SENS) and 1–3 µM (#16SENS); dose range for talazoparib: 0.1–0.3 µM (#1SENS and #19SENS) and 0.05–0.1 µM (#16SENS)). For each 1-week treatment round, after 72h-of treatment cells were dissociated as single cells and viable cells counted upon staining with Trypan Blue (#15250061, Thermo Scientific). In all experiments of neoR generation, no more than one round of 72h-treatment per week was performed to allow CRC-SCs to recover after each treatment. For insights on the two sets of #1neoR-CRC-SCs (#1neoR^a^ and #1neoR^b^) reported in Fig. 2e and Supplementary Fig. S4a see the Immunoblotting section.

### Measurement of cell proliferation/viability, generation of the drug sensitivity heatmap and analysis of drug synergisms

To determine the impact of single drugs or drug combinations on cell proliferation and survival, CRC-SCs dissociated at single cells were seeded in 96-well plates (100 µL of medium/well) at a density of 6 × 10^3^ cells/well. CRC-SCs were then cultured for 24 h in drug-free medium and left untreated or treated according to specific experimentations, as indicated in each figure and/or figure legend. Upon 72 h or 96 h from (co)treatment, cell viability/proliferation was measured by the CellTiter-Glo^®^ Luminescent Cell Viability Assay (#G7572, Promega, Madison, WI) based on luminescence counting of ATP levels by a multimode reader (DTX-880; Beckman Coulter, Brea, CA), according to the manufacturer’s instructions. All experiments were performed in triplicate parallel instances and the means of the triplicate (or duplicate in case of technical problems with one replicate, which were excluded from the analysis) were used for data representation (see Statistical procedures). The heatmap in Fig. 1e illustrates the log_2_ values of the ratio of percentage of viable neoR-CRC-SCs vs. viable SENS-CRC-SCs (neoR/SENS) upon drug administration calculated for each indicated dose using data from dose-response viability curves reported in Fig. 1 and Supplementary Figs. S1 and S2. Drug sensitivity heatmap was generated using the “R” software (R Foundation for Statistical Computing, Vienna, Austria; http://www.R-project.org/) and the following packages: ‘tcltk’, ‘tidyverse’, ‘readxl’, “ComplexHeatmap”, ‘reshape2’, and ‘data.table’. The scale of the heatmap is from −3 (green; meaning that SENS-CRC-SCs are more resistant than neoR-CRC-SCs) to 3 (red; meaning that neoR-CRC-SCs are more resistant than SENS-CRC-SCs). To improve heatmap readability and allow ratio calculation we applied the following changes. First, the percentage of viable cells used to calculate log_2_ ratio values was arbitrarily set to “1” in the following conditions in which this value was “0”: (i) 50 µM rabusertib and 50 µM CCT241533 for #1neoR; (ii) 50 µM rabusertib and 50 µM CCT241533 for #19SENS; and (iii) 50 µM rabusertib and 50 µM CCT241533 for #19neoR (see Fig. 1; Supplementary Fig. S1; Supplementary Table S2). Second, to generate the heatmap, the log_2_ value (#1neoR/#1SENS) of 12.5 µM and 50 µM berzosertib was set to the maximum value “3” (Supplementary Table S2). To calculate the synergistic drug effects in killing CRC-SCs, dose-response data were analyzed on the basis of the Loewe additivity model and visualized as a dose-response surface using the Combenefit software [74] (see also https://sourceforge.net/projects/combenefit/). In figures reporting the synergism, a multicolor code was used to easily identified drug combination effects, with synergy and antagonism depicted respectively in blue and red. Results were confirmed with the DrugComb Portal (https://drugcomb.fimm.fi/) also using the Bliss independence (BLISS), Highest Single Agent (HSA) and Zero interaction potency (ZIP) models. See [74] and [75] for primary references and further insights on the drug combination effect models employed.

### Clonogenic assay

For clonogenic potential assessment, CRC-SCs cultured as reported above were left untreated or exposed for 24 h (Fig. 1c) or 72 h (Figs. 5a, 6c, and 7b; Supplementary Figs. S5b, S6c, and S7c) to CHK1i, PARP1i, MRE11i (mirin) and/or RAD51i (B02) (first round of treatment). Upon treatment, CRC-SCs were washed (to remove the drug) and dissociated at single cells. Upon resuspension in 0.3% agarose (#50111, SeaPlaque GTG Agarose; Lonza), cells were seeded, at least in duplicate, in 24-well plates at a density of 500–1000 cells/well over a layer of 0.4% agarose. Experiments were always repeated at least in triplicate independent instances. Cells were then cultured for soft agar assays in drug-free CSC medium with the exception of Supplementary Fig. S5b in which they were treated again with specific drugs (second round of treament), and incubated under standard culture conditions for up to 14 days as reported in figure legends. Finally, samples were fixed/stained with 0.02% crystal violet (#C0775) and colonies counted. Only wells with countable colonies were considered in the analysis. The means of the replicates were used for data representation of each independent experiment (see Statistical procedures). For each CRC-SCs, representative images were selected from one or more clonogenic multi-well plates of the same experiment with similar results and, upon selection and crop from the original image, single wells were ordered in figure panels according to the following experimental conditions: control, 1st monotherapy, 2nd monotherapy, and combined therapy. Survival fractions were calculated by normalizing the number of colonies in treated conditions to the plating efficiency of untreated cells (i.e., colony formation over control condition). In Supplementary Fig. S5b quantification of colony formation for cells cultivated in drug-free condition in the first round of treatment (i.e., the first columns of the six group of two columns reported in the histogram) are a pool of data coming from 3 independent experiments some of which are also used for quantifying the same parameters in Fig. 5a. Only experiments including all experimental conditions are used for the quantification reported in Fig. 5a.

### Cytofluorometric studies

For the measurement of DNA content, γH2AX and/or pH3 levels (to assess DNA-damaged and mitotic cells, respectively), ethanol-fixed samples were permeabilized with 0.25% (v/v) Tween 20 (#P1379) in PBS at 4 °C for 15 min and, upon incubation in 3% (w/v) BSA in PBS for 30 min at 4 °C (to block unspecific binding), stained with a primary anti-γH2AX (S139) (1:250; #05-636, Merck Millipore; RRID:AB_309864) and/or a primary anti-pH3 (S10) (1:1500; #3377, Cell Signaling Technology, Danvers, MA; RRID:AB_1549592) in 1% (w/v) BSA in PBS overnight at 4 °C. Samples were then co-stained for 30 min at 4 °C with an Alexa Fluor^®^ 488-goat anti-mouse secondary antibody (#A-21121; RRID:AB_141514) and/or an Alexa Fluor^®^ 555-goat or 647-donkey anti-rabbit secondary antibody (#A-21429; RRID:AB_141761 or #A-31573; RRID:AB_2536183, respectively) (both 1:500; Thermo Scientific) together with 10 µM DAPI (1 mM; #D1306, Thermo Scientific) in 1% (w/v) BSA in PBS. To assess the impact of CHK1i on the expression of surface colorectal-CSC markers, 3×10^5^ cells seeded in 6-well plates (2 mL of medium/well) were cultured for 24 h, and then left untreated or exposed to 100 or 500 nM CHK1i. Upon 24 h or 48 h from treatment, cells were resuspended at 8 × 10^5^/mL, seeded in 96-well plates (100 µL of medium/well) and stained with a CD44v6-purified antibody (diluted 1:20 in CSC culture medium; #BBA13, R&D Systems, Minneapolis, MN; RRID:AB_356935). Samples were incubated in the dark and on ice for 30 min and, upon two washes with CSC culture medium, co-stained for 30 min with the appropriate Alexa Fluor^®^ 488-goat anti-mouse secondary antibody (1:500 in CSC culture medium). Cells were washed twice and then pellets were resuspended in 100 μL CSC culture medium supplemented with 1 µM DAPI. Cells were incubated overnight at 4 °C upon flow cytometry-mediated analyses. Cell cycle progression was assessed by cytofluorimetry as previously described [76]. Cytofluorometric acquisitions were performed by means of a BD FACSCanto^TM^ II (BD Biosciences, BD, Franklin Lakes, NJ), a Gallios^®^ Flow Cytometer (Beckman Coulter, Indianapolis, IN) or a MACSQuant^®^ Analyzer 10 (Miltenyi Biotec, Bergisch Gladbach, Germany) while data were statistically evaluated using the FlowJo software (FlowJo LLC, BD; RRID:SCR_008520). Only the events characterized by normal forward scatter (FSC) and side scatter (SSC) parameters were gated for inclusion in the statistical analysis. Surface colorectal-CSC markers were analyzed only on viable cells (*i.e*., cells excluding DAPI). Cell cycle analyses were performed upon gating on singlets and upon exclusion of the sub-G_1_.

### Hydroxyurea-nocodazole (HU + N) assay

To assess RSR functionality in basal conditions, we used a pre-validated and approved protocol based on the administration of 1 mM hydroxyurea (HU) for 15 h followed by drug washout and treatment with 1 µM nocodazole (N) for 24 h [42, 44, 45, 77]. In experiments aimed at analyzing the role of CHK1, MRE11, PARP1, and/or RAD51 on RSR functionality (Figs. 2b, 3c, and 7d; Supplementary Figs. S3a, S3b, and S4b), HU-treated CRC-SCs were washed and administered for 24 h with nocodazole together with inhibitors of CHK1 (100 nM CHK1i), MRE11 (20 µM mirin), PARP1 (500 nM talazoparib) and/or RAD51 (5 µM B02). In all cases, samples were recollected, fixed with ethanol and stained with the DNA dye DAPI together with an antibody directed against pH3 (S10) (1:1500; #3377) and/or γH2AX (1:250; #05-636) prior to cycle profiling analyses by flow cytometry as reported in the Cytofluorometric studies section. For the interpretation of the HU + N assay, the presence of a functional RSR allows cells to resolve RS induced by HU, and this results in DNA replication restart, S-phase progression and metaphase (and thus not S-phase) arrest when cells are challenged with nocodazole. On the contrary, cells with impaired RSR display an S-phase arrest, as shown by an increase in the fraction of cells with a DNA content between 2*n* and 4*n* in cell cycle profiles. This is due to the accumulation of ssDNA from unresolved RS, whose entity depends on the severity of RSR impairment. Note that the quantification of the percentage of S-phase, G_2_/M-phase and pH3^+^ cells in Supplementary Fig. S3a and of γH2AX^+^ cells among G_2_/M-phase in Supplementary Fig. S3b for #1SENS-CRC-SCs and #1neoR-CRC-SCs subjected to the HU + N protocol includes data from at least 4 independent experiments of which some are used for quantifying the same parameters in Figs. 3c and 7d. Only experiments including all experimental conditions are used for the quantification reported in Figs. 3c and 7d.

### Immunofluorescence

The immunofluorescence detection of DNA damage markers and SAC activation was performed as follow. Briefly, dissociated CRC-SCs were fixed in 4% (w/v) paraformaldehyde (PFA; #28908, Thermo Scientific) in PBS and deposited on poly-D-lysine-coated (#P7886) glass slides. Slides were air-dried and then permeabilized for 15 min with 0.1% (v/v) Triton X-100 (Amersham Biosciences; GE Healthcare Life Sciences, Little Chalfont, UK) in PBS. Samples were then incubated for 30 min in 5% (w/v) BSA in PBS and immunostained overnight at 4 °C or 2 h at RT with the following primary antibodies (diluted as indicated): bromodeoxyuridine (BrdU, 1:25; #347580; RRID:AB_400326), BUBR1 (1:50; #612502; RRID:AB_399803) (both from BD Biosciences), cleaved caspase-3 (1:400; #9661, Cell Signaling Technology; RRID:AB_2341188), rabbit γH2AX (S139) (1:400; #9718, Cell Signaling Technology; RRID:AB_2118009), mouse γH2AX (S139) (1:250; #05-636), pH3 (S10) (1:1500; #3377), pRPA32 (S4/S8) (1:250; #A300-245A, Bethyl Laboratories; RRID:AB_210547). The slides were finally incubated with the appropriate Alexa Fluor conjugates (1:600) and 10 µM Hoechst 33342 (to counterstain nuclei, #H1399) (both from Thermo Scientific). Fluorescence images were captured using a Leica DMR fluorescence microscope equipped with a 100X oil immersion objective and the Leica QWin software or a Leica DMI3000 B fluorescence microscope equipped with a 40X objective, Leica DFC 310FX camera and LAS X software (Leica Microsystems, Wetzlar, Germany). Analysis was performed directly by microscopy on 100 cells per condition and/or using Adobe Photoshop CC 2015 (Adobe, San Jose, CA; RRID:SCR_014199) and ImageJ v1.8 software (National Institute of Health, Bethesda, MD; http://rsb.info.nih.gov/ij/; RRID:SCR_003070). Cells were considered positive for γH2AX, pRPA32 and ssDNA when they display more than 5 visible/overlapping foci (Figs. 2d and 3g; Supplementary Fig. S4c). Note that in Fig. 2d the quantification of the percentage of pRPA32^+^ cells for neoR-CRC-SCs left untreated or treated with CHK1i includes data from at least 3 independent experiments of which some are used for quantifying the same parameters in Supplementary Fig. S4c. Spindle assembly checkpoint activation was assessed by evaluating nuclear/kinetochore localization of BUBR1 in metaphase cells (*i.e*., positive for pH3, pH3^+^) (Fig. 7g). The anaphase ratio reported in Fig. 7g was calculated by counting the fraction of anaphases on 100 prophases+metaphases+anaphases according to their classic morphology (via Hoechst 33342 staining) or pH3 positivity (prophases and metaphases: pH3^+^; anaphases: pH3^−^). To assess apoptosis induction, CRC-SCs were resuspended in 90% CSC culture medium/10% Matrigel Basement Membrane Matrix (#354230, Corning) to a final concentration of 3.5 × 10^5^ cells in 500 µL, and then seeded in 24-well plates. Upon 24 h, CRC-SCs were left untreated or exposed for 48 h to MRE11i and/or RAD51i alone or in combination with Q-VD-Oph or GSK’872. Finally, the cells were stained with 1 µg/mL propidium iodide (PI, a vital dye; #P3566, Thermo Scientific) and 8 µM Hoechst 33342 and analyzed by live fluorescence microscopy. Images were captured using a Leica DMI3000 B fluorescence microscope equipped with a 20X objective, Leica DFC 310FX camera and LAS X software (Leica Microsystems). Representative images of at least two independent experiments with similar results are reported in Supplementary Fig. 8.

### Immunoblotting

The detection of protein levels was performed as previously reported [21]. Briefly, CRC-SCs were harvested, washed with PBS, and lysed for 30 min on ice with a buffer containing T-PER reagent (#78510, Thermo Scientific), 300 mM NaCl (#31434), and protease/phosphatase inhibitors cocktails (#P8340, #P5726, #P0044). Samples were then centrifuged for 15 min at 13000 rpm, and supernatants collected. Total protein concentration was determined by the Bradford reagent method (Bio-Rad Protein Assay, #5000002, Bio-Rad). Equal amounts of proteins (30 μg) were resolved by SDS-polyacrylamide gel electrophoresis (SDS-PAGE) and electro-transferred to a nitrocellulose membrane (#88018, Pierce-Thermo Scientific). Membranes were incubated overnight with the primary antibody of interest and then for 1 h at RT with the appropriate horseradish peroxidase-conjugate secondary antibody (sheep anti-mouse IgG whole antibody, #GENA931; RRID:AB_772210; donkey anti-rabbit IgG whole antibody, #GENA934; RRID:AB_2722659 or AB_772206; or mouse anti-goat IgG whole antibody, #sc-2354, Santa Cruz Biotechnology, Dallas, TX; RRID:AB_628490). Chemiluminescence 16-bit imaging was performed with the Kodak Image Station 4000R (IS4000R; Eastman Kodak Company, Rochester, NY) and the Carestream Molecular Imaging Software version 5.0 (Carestream Health, Rochester, NY), or with the G:Box Chemi-XX9 and GeneSys Software v1.5.6 (Synoptics, Cambridge, UK; RRID:SCR_015770). The following primary antibodies were used (diluted as indicated): pATM (S1981) (1:1000; #ab81292; RRID:AB_1640207), ATM (1:500; #ab78; RRID:AB_306089) (both from Abcam, Cambridge, UK), pCHK1 (S317) (1:1000; #12302; RRID:AB_2783865), CHK1 (1:750; #2345; RRID:AB_10693648), rabbit γH2AX (S139) (1:1000; #9718), PARP1 (1:1000; #9542; RRID:AB_2160739) (all from Cell Signaling Technology), pRPA32 (S4/S8) (1:1000; #A300-245A; RRID:AB_210547), and RPA32 (1:1000; #A303-874A; RRID:AB_2620224) (both from Bethyl Laboratories, Montgomery, TX). Anti-β-Actin (1:1000; #4967, Cell Signaling Technology; RRID:AB_330288), anti-C23/Nucleolin (1:1000; #sc-8031, Santa Cruz Biotechnology; RRID:AB_670271), anti-Cofilin (1:1000; #3318, Cell Signaling Technology; RRID: AB_2080595) or anti-β-Tubulin (1:1000; #T4026; RRID:AB_477577) antibodies were used to monitor equal loading of lanes. Representative western-blot images of at least two independent experiments with similar results are reported in figures, with the exception of Fig. 2e and Supplementary Fig. S4a in which one western-blot was performed. Indeed, in these figures, #1neoR^a^ and #1neoR^b^ correspond to cells recollected at different times during the protocol of generation of resistance to CHK1i, which last some months reducing the possibility to have replicates. Specifically: #1neoR^a^ after 12 weeks and #1neoR^b^ after 17 weeks from the first round of CHK1i administration. Of note, #1neoR^b^ display higher resistance to CHK1i than #1neoR^a^.

### Deep DNA sequencing

For deep DNA sequencing targeting of genes involved in the DDR (see Supplementary Table S3), we designed a custom panel using the QIAseq Targeted DNA Custom Panel Builder (Qiagen, Hilden, Germany). The libraries were prepared with 40 ng genomic DNA using the QIAseq targeted DNA technology following the manufacturer’s instructions. The resulting libraries were sequenced on an Illumina NextSeq 500 (Illumina, San Diego, CA) in paired-end mode, sequencing from each side 150 bp. FASTQ files were analyzed with CLC Genomics Workbench software (v12.0, Qiagen). The QIASeq Panel analysis was set on Somatic mode for Illumina reads. Variant annotation was carried out with Annovar (version update at July 2018) [78]. Only somatic variants with Allele Frequency>10, coverage>100x, Protein Damaging consequence, and dbSNP MAF < 5% were kept. Germline pathogenic variants were annotated with ClinVar [79] and pathogenicity of BRCA^*^ variants was annotated via the BRCA Exchange database (https://brcaexchange.org/) [80]. The Oncoprint (Fig. 3a) was generated using custom R scripting and the ComplexHeatmaps library available at https://github.com/jokergoo/ComplexHeatmap. For the Oncoprint, mutations were further filtered on the basis of the following criteria: (ì) annotated as (likely) oncogenic in oncoKB database (https://oncokb.org/) [81] and/or (ii) annotated on the COSMIC database (http://cancer.sanger.ac.uk/cosmic) (see Supplementary Table S3; Analysis performed in July 2019). Sequencing data generated in the context of this study have been deposited at European Genome-phenome Archive under accession numbers EGAS00001004892.

### DNA fiber assay

For DNA fiber length measurement, 4 × 10^5^ CRC-SCs were seeded as single cells in 6-well plates and, upon 24 h of cultivation, left untreated or treated for 6 h with CHK1i (100 and 200 nM for SENS-CRC-SCs and neoR-CRC-SCs, respectively) and/or talazoparib (500 nM). DNA replication sites were labeled with 250 µM 5-Iodo-2-deoxyuridine (IdU; #I7125) 30 min prior to the end of treatments. Cells were then washed with cold PBS and resuspended in 20–50 μl of cold PBS. DNA spreading was performed as follow: 2.5 μl of each cell suspension was spotted at the end of the microscope slide (SuperFrost slides; VWR international, Radnor, PA) and air-dried for a few minutes. Thereafter 7.5 μl of the lysis solution (200 mM Tris-HCl-pH 7.5, 50 mM EDTA, 0.5% SDS in water) was applied on top of the cell suspension, then mixed by gently stirring with a pipette tip and incubated for 1 min 45 s. The slides were tilted to 15° to allow the DNA fibers spreading along the slide. The dried DNA fibers were then fixed in a methanol/acetic acid (3:1) solution for 10 min, washed in dH_2_O, and denaturated in 2.5 M HCl for 80 min. Labeled DNA fiber immunodetection was performed via sequential incubation of the slides for 20 min with a blocking solution (5% BSA in PBS), for 2 h at RT with a primary antibody solution (anti-BrdU diluted 1:25), and for 1 h at RT with the appropriate secondary antibody solution. Images were acquired randomly from fields with untangled fibers using the Eclipse 80i Nikon Fluorescence Microscope (Nikon, Minato-ku, Tokyo, Japan), equipped with a VideoConfocal (ViCo) system (Nikon). Labeled tract lengths were measured using ImageJ v1.8 software. A minimum of 191 DNA fibers pooled from two or four independent experiments were analyzed for each experimental condition. Only well-isolated DNA fibers were considered in the analysis. In Fig. 3f, the dot plot on the left (track length comparison between untreated SENS-CRC-SCs vs. untreated neoR-CRC-SCs) reports data pooled from four experiments, including two experiments which are also reported in the dot plots concerning SENS-CRC-SCs (in the center) and neoR-CRC-SCs (on the right).

### Detection of single-stranded DNA by native IdU assay

To detect nascent ssDNA, 3 × 10^5^ CRC-SCs were seeded as single cells in 6-well plates and, upon 24 h cultivation, were either left treated or treated with specific pharmacological inhibitors. Finally, cells were administrated with 250 µM IdU for 30 min before the end of treatments as reported in [49]. Alternatively, to detect parental ssDNA, upon 24 h cultivation, seeded cells were administrated with 10 µM IdU for 24 h as indicated in [49]. IdU-stained cells were then left untreated or exposed to CHK1i (200 nM), and/or talazoparib (500 nM) for 6 h. In both protocols, cells were dissociated and fixed with 4% PFA. Finally, nascent/parental ssDNA was immunodetected by using a primary antibody directed against BrdU, as described in the Immunofluorescence section. Quantitative nascent DNA analyses were performed on ≥12 randomly selected images. Briefly, blue nuclear fluorescence was used to identify nuclear regions on which green intensity (nascent DNA) means were quantified. All steps were performed using ImageJ v1.8 software. A minimum of 509 cells per condition pooled from two independent experiments were analyzed. Quantitative parental DNA analyses was performed as reported in Immunofluorescence section.

### In vivo study

In vivo studies are all included in an experimental protocol approved by the Institutional Animal Experimentation Committee (authorization no. 08/2017-PR and 227/2016-PR). Housing and handling of animals were in strict compliance with national Animal Experimentation Guidelines for Laboratory Animal Welfare (D.L.26/14) and in accordance with the Directive EU63/2010. Female NOD.Cg-Prkdcscid Il2rgtm1Wjl/SzJ (NSG; #005557; RRID: IMSR_JAX:005557) mice of 4–6-week-old (~21 g) were provided from The Jackson Laboratory (Bar Harbor, ME). Animals were always employed after an acclimatization period of 14 days and left under ad libitum feeding and drinking conditions. For mice engraftment, CRC-SCs were resuspended in 50% CSC culture medium/50% Matrigel Basement Membrane Matrix to a final concentration of 5 × 10^6^ cells/mL, and then 5 × 10^5^ cells were injected subcutaneously in the flank of mice. Tumors were measured twice a week by a common caliper. When tumors were palpable (i.e., ~14 mm^3^, 2–3 weeks from the inocolum), mice were randomly allocated to the following treatment groups: Control (vehicles), CHK1i-treated, olaparib-treated and CHK1i + olaparib co-treated groups. No blinding was performed. In more detail, CHK1i dissolved in 50% H_2_O and 50% of a solution of 40% Cyclodextrin (#OC15979, Carbosynth, Compton, UK) was administered at a dose of 5 mg/kg for three cycles of 1-week consisting of three consecutive days, *bis in die*, followed by 2 days of rest + 1 day of treatment (*bis in die*) and one day of rest, while olaparib dissolved in 11,6% DMSO + 25% of a solution of 40% Cyclodextrin + 63.4% PBS was administered at a dose of 50 mg/kg for three cycles of three consecutive days, once a day, followed by 2 days of rest + 1 day of treatment (once a day) and one day of rest. To calculate tumor volumes, we used the modified ellipsoidal formula: V = (length × width^2^)/2. At least 7 mice per group were employed (see Statistical procedures). Outliers or mice with symptoms non attributable to cancer were excluded as a predetermined criterion. Animals were euthanized according to the national Animal Welfare Guidelines. See [21] for further details.

### Statistical procedures

For in vitro experiments, no statistical methods were used to determine sample size. In vitro experiments were independently repeated at least three times with similar results, with the exception of few cases in which experiments were repeated less than three times (always specified in figure legends and/or in specific Materials and Methods sections). In case of lack of clarity or conclusiveness in statistical sub-significant trends, sample number was increased to more than 3 to improve statistical power. In in vivo experiments, sample size was determined as follows. Considering as outcome of interest the reduction of tumor growth upon treatment, we hypothesized that 1% of tumors in the group of mice treated with the vehicle and 70% of tumors in the group of mice treated with PARP1i + CHK1i display a decrease in their in vivo growth. Applying a one-sided Fisher’s Exact test and considering a statistical power equal to 80% and a significance level of 0.05, we used at least 7 mice for all groups. The exact sample size (*n*) for each experimental group/condition, whether the samples represent technical or biological replicates, the number of replicates in individual experiments, and data/replicate exclusion criteria are reported in figure legends or in the specific Materials and Methods section. Data were analyzed with Microsoft Excel (Microsoft, Redmond, WA) or Prism (v8.3.0, GraphPad Software, San Diego, CA), while statistical analyses were carried out using Prism and SPSS software (SPSS v.21, SPSS Inc-IBM, Chicago, IL). For each set of data of each in vitro experiment conducted at least in three independent instances, normal distribution was controlled with the Shapiro–Wilk test using SPSS and/or Prism. In case of data with a normal distribution, statistical analysis was performed as follow. For comparisons involving only two groups of samples, statistical significance was evaluated by unpaired t-test or Welch’s unpaired t-test, depending on variance equality between the two groups (compared using the F-test). For comparisons involving more than two groups of samples, statistical significance was evaluated by ordinary one-way ANOVA followed by Bonferroni post-hoc test or by Brown–Forsythe and Welch one-way ANOVA followed by Dunnett T3 post-hoc test depending on variance equality (assessed using the Brown–Forsythe test). Alternatively, in case of data not normally distributed or of two independent experiments, Mann−Whitney test or Kruskal−Wallis test followed by Dunn’s post-hoc test was applied. Statistical significance on in vivo growth curves was assessed by two-way ANOVA followed by Bonferroni post-hoc test. For the last time point, statical significance of tumor growth variation was evaluated with Kruskal−Wallis test followed by Dunn’s post-hoc test. In the dose-response curves of Fig. 1, statistical analysis was not performed in few conditions in which, in at least one CRC-SCs, a very low percentages of cells (less than 2%, mean of ≥3 independent experiments) survived the treatment, as for the highest concentration(s) of berzosertib (12.5 and 50 µM), camptothecin (12.5 and 50 µM), prexasertib (50 µM) and rabusertib (50 µM). Statistical analysis for γH2AX^+^ S-phase or G_2_/M-phase cells was not performed given the low percentage (≤3%) of positive cells (Supplementary Fig. S7g). We considered statistically significant *P* values less than 0.05. All significant *P* values are reported in Supplementary Table S4. In in vitro experiments involving normalization of treated on untreated conditions, controls are expressed as percentages ± SEM calculated upon normalization on the average of raw control data of all experiments included in each analysis. In experiments with small sample sizes (*n* < 5), individual data points from independent experiments are represented as single dots either in box-plots in which also the mean is illustrated or in columns representing mean ± SEM. For figure readability, experimental data of dose-response curves of Fig. 1b and 1c are shown as mean ± SEM, while individual experiments for each drug treatment are reported in Supplementary Figs. S1c and S1e. Likewise, in Fig. 6a and 6e, data are reported as mean ± SEM calculated from the individual experiments illustrated in Supplementary Figs. S6b and S6f, with SEM for control condition calculated as described above and statistical analysis performed only for the last time point.

## Supplementary information

Supplementary information_Manic et al_CDD-20-1279.RR.pdf

Supplementary Figures

Supplementary Table S1_Manic et al_CDD-20-1279.RR

Supplementary Table S2_Manic et al_CDD-20-1279.RR

Supplementary Table S3_Manic et al_CDD-20-1279.RR

Supplementary Table S4_Manic et al_CDD-20-1279.RR
