# Supplementary material for: Control of replication stress and mitosis in colorectal cancer stem cells through the interplay of PARP1, MRE11 and RAD51
Source: Cell Death Differ. 2021 Feb 2;28(7):2060–82. doi: 10.1038/s41418-020-00733-4 (PMC8257675; doi:10.1038/s41418-020-00733-4)
Supplement: Supplementary file 1 — Supplementary information_Manic et al_CDD-20-1279.RR.pdf [file 41418_2020_733_MOESM1_ESM.pdf]

Legends to Supplementary Figures

Supplementary Figure S1. Related to Figure 1.

Impact of DNA damage response inhibitors and RS inducers on CRC-SC survival.

(**a**) Classification of our panel of primary CRC-SCs according to their sensitivity or resistance to the CHK1 inhibitor prexasertib (CHK1i) based the IC_{50} reported in [1].

CHK1-resistant CRC-SCs (innR-CRC-SCs) display an IC_{50} > 500nM at both 48h and 72h of treatment.

Among CHK1-sensitive (SENS) CRC-SCs:

- hypersensitive ($SENS^{HIGH}$) CRC-SCs display an IC_{50} < 100nM at both 48h and 72h of treatment.
- moderately sensitive ($SENS^{MED}$) CRC-SCs display a IC_{50} > 100nM at 48h of treatment and < 500nM at 72h of treatment.

(**b**) NeoR-CRC-SCs (#19neoR) were left untreated or administered with CHK1 inhibitor prexasertib (CHK1i) as depicted, and then costained with an antibody directed against the CRC-SC marker CD44v6 and the vital dye DAPI followed by cytofluorimetric analysis. Representative biparametric plots (numbers indicate the percentages of corresponding events) as well as a histogram reporting quantitative data of the fold change decrease of $CD44v6^{+}$ cells as compared to control conditions from two independent experiments are reported. Only viable cells (*i.e.*, cells excluding DAPI) were included in the analysis. No statistical differences between treated and untreated conditions were observed (Kruskal-Wallis ANOVA and Dunn’s post-hoc test).

(**c**) Individual dose-response curves of neoR-CRC-SCs *vs.* SENS-CRC-SCs left untreated or exposed for 72h to CHK1i at the indicated dose-range (from 49nM to 50µM). Proliferation/viability was assessed by CellTiter-Glo® assay. Each curve is an independent experiment. Dose response curve calculated from these individual dose-response curves and expressed as means±SEM are shown in **Fig. 1b**.

(**d**) NeoR-CRC-SCs (#1neoR and #19neoR) were left untreated or treated with CHK1i at the indicated dose range (from 49nM to 50µM) in the presence or absence of the inhibitor of the P-glycoprotein efflux pump Verapamil at 10µM. After 72h, cell proliferation/viability was evaluated by means of CellTiter-Glo® assay. Individual dose response curves from 3 independent experiments together with the dose response curve calculated from these individual curves and expressed as means±SEM are reported. No statistical differences were observed between conditions treated with CHK1i + Verapamil or treated only with CHK1i (unpaired t-test with or without Welch's correction was applied).

(**e**) Individual dose-response curves of neoR-CRC-SCs *vs.* SENS-CRC-SCs left untreated or exposed for 72h to the indicated ATR inhibitor, CHK1 inhibitor or irinotecan, a representative RS inducer. Proliferation/viability was assessed by CellTiter-Glo® assay. Each curve is an independent experiment. Dose response curves from these individual curves with data expressed as means±SEM are shown in **Fig. 1d**.

(**f**) Both pairs of neoR-CRC-SCs and SENS-CRC-SCs were left untreated or treated with pharmacological inhibitors of the DNA damage response kinase ATM (KU-55933, KU-60019), ATR (VE-821), CHK2 (CCT241533, PV1019), DNA-PK (NU7026), or clinical relevant poisons 5-fluorouracil (5-FU) and oxaliplatin at the indicated dose range (from 49nM to 50µM). After 72h, cell proliferation/viability was evaluated by means of CellTiter-Glo® assay. For each drug, the panel reports both the individual dose response curves coming from the illustrated number of independent experiments (on the right) and the dose response curves calculated from these individual curves and expressed as means±SEM (on the left). $^{*}P$<0.05, $^{**}P$<0.01, $^{***}P$<0.001 (unpaired t-test with or without Welch's correction or Mann-Whitney test) as compared to the corresponding neoR-CRC-SCs treated with the same inhibitor at the same dose. Refer also to **Fig. 1e**.

All significant *P* values are shown in **Supplementary Table S4**.

Supplementary Figure S2. Related to Figure 1

NeoR-CRC-SCs *vs.* SENS-CRC-SCs were left untreated or treated with pharmacological inhibitors of the DNA damage response (B02, mirin, NP-004255, olaparib, talazoparib, rucaparib, and veliparib) or inducers of replication stress (adavosertib, camptothecin, cisplatin, etoposide, gemcitabine, and triapine) at the indicated dose range (from 49nM to 50μM). After 72h, cell proliferation/viability was evaluated by means of CellTiter-Glo® assay. For each drug, the panel reports both the individual dose response curves coming from the illustrated number of independent experiments (on the right) and the dose response curves calculated from these individual curves and expressed as means±SEM (on the left). $^{*}P<0.05$, $^{**}P<0.01$, $^{***}P<0.001$ (unpaired t-test, unpaired t-test with Welch's correction or Mann-Whitney test) as compared to the corresponding neoR-CRC-SCs treated with the same inhibitor at the same dose. Refer also to **Fig. 1e**.

All significant *P* values are shown in **Supplementary Table S4**.

Supplementary Figure S3. Related to Figure 2.

Impact of CHK1 inhibitors on cell cycle progression and RS levels of CRC-SCs.

(**a,b**) NeoR-CRC-SCs and SENS-CRC-SCs were left untreated, or treated with 1mM hydroxyurea (HU) for 15h, followed by drug washout and treatment for 24h with 1μM nocodazole (N) alone (the HU+N protocol) (*see* **Materials and Methods**). Cells were then fixed with cold ethanol and costained with the DNA dye DAPI and antibodies directed against phospho-histone H3 (pH3) (**a**) and phospho-H2AX (γH2AX) (**b**) before cytofluorimetric analysis of DNA content, DNA damage and mitosis. Panels report the quantification of the percentage of S-phase cells, G_2/M-phase cells and $pH3^{+}$ cells in mitosis (**a**) and of $\gamma H2AX^{+}$ cells among G_2/M-phase cells (**b**) from cell cycle profiles and data of **Fig. 2b**. Results in **a** are expressed as means±SEM and individual data points from the reported number of independent experiments. Results in **b** are expressed as means±SEM of 8 (SENS-CRC-SCs) and 11 (neoR-CRC-SCs)

independent experiments. No significant differences were observed between the depicted treatment conditions (**a**, one-way ANOVA and Bonferroni post-hoc test; **b**, unpaired t-test).

(**c**) NeoR-CRC-SCs and SENS-CRC-SCs were left untreated or administered for 24h with 100nM CHK1 inhibitor prexasertib (CHK1i) or gemcitabine (GEM). Cells were then fixed with cold ethanol and costained with the DNA dye DAPI and antibodies directed against γH2AX prior to cytofluorimetric assessment of DNA content and DNA damage. The panel reports representative cell cycle profiles (with numbers indicating the percentages of corresponding events) and the quantification of the percentages of γH2AX$^+$ cells among S-phase cells expressed as means±SEM of 6 (SENS-CRC-SCs) and 8 (neoR-CRC-SCs) independent experiments. $^*P<0.05$, $^{**}P<0.01$, $^{***}P<0.001$ (Welch's unpaired t-test) as compared to the corresponding CRC-SCs left untreated. *See* also **Fig. 2c**.

(**d**) The illustrated CRC-SCs were left untreated (-) or administrated for 6h or 24h with 100nM of CHK1i, and then subjected to western-blot analyses with antibodies directed against the phosphorylated (p) or total form of RPA32 or against γH2AX (see also **Fig. 2e**). Nucleolin was used as equal loading control.

(**e**) NeoR-CRC-SCs (#1neoR and #19neoR) were left untreated (control, CTR) or subjected to 24h-treatment with 100nM CHK1i. Cells were then fixed, costained with an anti-pH3 antibody and DAPI before cytofluorimetric analysis of DNA content and mitotic cell fraction. Representative cell cycle profiles are shown together with quantitative data (means±SEM) concerning the percentage of S-phase cells (5 independent experiments), the fold change of pH3$^+$ cells as compared to control conditions (4 independent experiments) and the percentage of premature mitosis - *i.e.*, pH3$^+$ cells with a <4*n* DNA content, as compared to normal mitosis, *i.e.*, pH3$^+$ cells with a 4*n* DNA content - (4 independent experiments). The sub-G_1 fraction was excluded from the analysis. No significant differences were observed between the depicted treatment conditions (left, Kruskal-Wallis test and Dunn’s post-hoc test; center-right, one-way ANOVA and Bonferroni post-hoc test).

(**f-h**) Both neoR-CRC-SCs were left untreated or treated with CHK1i (**f,g**) or the inhibitor of ATR VE-821 (**h**) alone or in combination with the indicated doses of RS inducers. CRC-SC proliferation/viability was determined 72h later by means of CellTiter-Glo® assay. Data from both neoR-CRC-SCs were pooled. Results are presented as means±SEM of 5 (**f**, gemcitabine; **h**, gemcitabine) or 6 (**f**, irinotecan) independent experiments or as means±SEM and individual data of 3 independent experiments (**g**, adavoseritib and triapine, with the exception of untreated and 500nM CHK1i-treated cells in the experiments assessing adavoseritib, in which 4 independent experiments were performed). $^{*}P<0.05$, $^{**}P<0.01$, $^{***}P<0.001$ (**f-h**, one-way ANOVA and Bonferroni or Dunnett T3 post-hoc test; **g**, triapine, unpaired t-test) as indicated.

All significant *P* values are shown in **Supplementary Table S4**.

Supplementary Figure S4. Related to Figure 3.

Impact of PARP1 on the RSR of CRC-SCs.

(**a**) #1neoR-CRC-SCs *vs.* #1SENS-CRC-SCs were left untreated (-) or exposed for 6h or 24h to prexasertib (CHK1i) and then subjected to western-blot analyses using antibodies recognizing PARP1 or β-Tubulin to ensure equal loading of lanes. Note that the two sets of #1neoR-CRC-SCs correspond to cells recollected at different times during the protocol of generation of resistance to CHK1i (#1neoR^{a}, after 12 weeks from the first CHK1 administration round; #1neoR^{b} after 17 weeks from the first CHK1 administration round), and that #1neoR^{b} display higher resistance to CHK1i than #1neoR^{a}. PARP1 levels in established #1neoR-CRC-SCs (25 weeks) are shown in **Fig. 3b**. cPARP1, cleaved PARP1.

(**b**) NeoR-CRC-SCs and SENS-CRC-SCs (#1SENS/neoR) either were left untreated or treated for 15h with 1mM hydroxyurea (HU) and, after a PBS-wash, for 24h with 1µM nocodazole (N) alone or together with 100nM of CHK1i and/or 500nM of the PARP1 inhibitor (PARP1i) talazoparib (the HU+N assay, *see* **Materials and Methods**). After cell fixation in ethanol, cells were costained with DAPI and

antibodies against γH2AX and phospho-histone H3 (pH3) for flow cytometry-mediated assessment of DNA content, DNA damage and mitosis. Cell cycle profiles with the indicated DNA content and the percentage of mitosis ($pH3^+$ cells in G_2/M-phase, depicted in red) are shown. Numbers indicate the percentages of corresponding events. For cell cycle profiles of neoR-CRC-SCs and quantitative data refer to **Fig. 3c**. Quantitative data report the percentage of $\gamma H2AX^+$ cells among G_2/M phase cells (means±SEM; n=3 independent experiments). $^{*}P<0.05$, $^{**}P<0.01$, $^{***}P<0.001$ (one-way ANOVA and Bonferroni post-hoc test), as indicated. $^{\#}P<0.05$, $^{\#\#}P<0.01$, $^{\#\#\#}P<0.001$ (unpaired t-test), as indicated.

(**c**) Fluorescence microscopy analysis of RS markers in neoR-CRC-SCs left untreated or treated with CHK1i and/or PARP1i (talazoparib) for 24h and stained with antibodies recognizing γH2AX or pRPA32. Quantification of $\gamma H2AX^+$ or $pRPA32^+$ cells (>5 foci) are reported. Results are means ± SEM and individual data points from 5 (γH2AX staining) or 3 (pRPA32 staining) independent experiments $^{*}P<0.05$, $^{**}P<0.01$, $^{***}P<0.001$ (one-way ANOVA and Bonferroni post-hoc test), as indicated.

All significant *P* values are shown in **Supplementary Table S4**.

Supplementary Figure S5. Related to Figures 4 and 5.

Impact of PARP1 inhibition in CRC-SC sensitivity to CHK1i.

(**a**) Cell viability (assessed by CellTiter-Glo® assay) of neoR-CRC-SCs left untreated (-) or subjected to 2 consecutive rounds of 72h treatments (+) with 100nM prexasertib (CHK1i) and/or the PARP1i inhibitor olaparib (OLA, 5µM) or talazoparib (TZ, 300nM) separated by drug washes, as indicated and reported in **Materials and Methods**. Results are means±SEM and individual data points from 3 independent experiments. Statistical analysis: one-way ANOVA and Bonferroni post-hoc test; $^{*}P<0.05$, $^{**}P<0.01$, $^{***}P<0.001$ as compared to the corresponding CRC-SCs left untreated. *See* also **Fig. 4a**.

(**b**) Clonogenic survival of neoR-CRC-SCs pretreated for 72h with 100nM CHK1i alone or together with 5µM OLA or 300nM TZ (first round of treatment) and then cultivated in drug free medium (-) or in the

presence (+) of CHK1i and/or PARP1i for up to 15 days (second round of treatment) as indicated. Representative images and quantitative data (pool of data from #1neoR and #19neoR) are reported. Results are means±SEM and individual data points from 3 independent experiments. $^{*}P<0.05$, $^{**}P<0.01$, $^{***}P<0.001$ (one-way ANOVA and Bonferroni post-hoc test), as compared to untreated conditions. See also **Fig. 5a**.

All significant *P* values are shown in **Supplementary Table S4**.

Supplementary Figure S6. Related to Figure 6.

Impact of PARP1 on the development of CHK1i resistance in CRC-SCs.

(**a**) Scheme of the experimental protocol used to assess the impact of PARP1 inhibition on the generation of neoR-CRC-SCs. SENS-CRC-SCs were left untreated (not shown) or subjected to consecutive rounds of 72h-treatment with prexasertib (CHK1i) alone or in combination with PARP1 inhibitor (PARP1i) followed by ≥4 days of cultivation in drug-free medium, as described in **Materials and Methods**. *See* **Fig. 6a**,**e**.

(**b**) Cell viability of SENS-CRC-SCs treated as indicated in **Materials and Methods** and in panel **a**. After each round of treatment, viable cells were counted upon Trypan Blue staining. Three individual independent experiments from which **Fig. 6a** is derived are reported. Doses employed: CHK1i 1-30nM; olaparib (OLA) 2-7µM; talazoparib (TZ) 100-500nM.

(**c**) Clonogenic survival of SENS-CRC-SCs left untreated or pre-treated for 72h with 10nM CHK1i, 5µM OLA and/or 300nM TZ. Representative images are shown. See also quantitative data in **Fig. 6c**.

(**d**) SENS-CRC-SCs (#1SENS and #19SENS) were left untreated or treated for 72h with sublethal doses of CHK1i alone or in combination with the WEE1 inhibitor adavosertib or the RRM2 inhibitor triapine before assessment of cell proliferation/viability by means of CellTiter-Glo® assay. Data from #1SENS

and #19SENS were pooled (means±SEM and individual data points; n=4 independent experiments). $^{*}P<0.05$, $^{**}P<0.01$, $^{***}P<0.001$ (unpaired t-test or Welch's unpaired t-test) as indicated.

(**e**) Six representative CRC-SCs, 3 $SENS^{HIGH}$ (#16, #18, #30) and 3 $SENS^{MED}$ (#3, #6, #29), were rendered resistant to CHK1 inhibition (neo-resistant, neoR; *see* **Fig. 6d**) by using the protocol described in **Materials and Methods** and applied in **Fig. 1a**. These neoR-CRC-SCs as well as their parental SENS-CRC-SCs counterparts were left untreated or treated with CHK1i for 72h at the indicated dose-range (from 49nM to 50µM). Cell proliferation/viability was then evaluated by means of CellTiter-Glo® assay. Individual dose-response curves, dose response curves derived from them and expressed as means±SEM, and number of independent experiments are reported.

(**f**) Cell viability of SENS-CRC-SCs treated as indicated in **Materials and Methods** and in panel **a**. After each round of treatment, viable cells were counted upon Trypan Blue staining. Three individual independent experiments from which **Fig. 6e** is derived are reported. Doses employed: CHK1i 1-30nM; OLA 2-7µM; TZ 100-500nM.

All significant *P* values are shown in **Supplementary Table S4**.

Supplementary Figure S7. Related to Figure 7.

Impact of MRE11 and RAD51 on the RSR, cell division and survival of CSCs.

(**a,b**) Cell viability (assessed by CellTiter-Glo® assay) of the reported neoR-CRC-SCs exposed to the MRE11 inhibitor mirin (MRE11i) and/or the RAD51 inhibitor B02 (RAD51i) for 96h (**a**,**b**) as indicated. Results are means±SEM from 5 independent experiments. $^{*}P<0.05$, $^{**}P<0.01$, $^{***}P<0.001$ (one-way ANOVA and Bonferroni post-hoc test), as indicated. In **b**, synergism is calculated using the Combenefit software (see **Materials and Methods**). See **Fig. 7a**.

(**c**) Clonogenic survival of the reported neoR-CRC-SCs exposed to MRE11i and/or the RAD51i for 72h as indicated. Representative images and quantitative data are shown (dose range: #1neoR: 20µM

MRE11i, 5μM RAD51i). Results are means±SEM and individual data points from 3 independent experiments. $^{*}P<0.05$, $^{**}P<0.01$, $^{***}P<0.001$ (one-way ANOVA and Bonferroni post-hoc test), as indicated.

(**d**,**e**) Cell viability (evaluated by CellTiter-Glo® assay) of neoR-CRC-SCs (**d**) and #1SENS and #19SENS and other CRC-SCs with moderate (SENS^{MED}; #3, #6) or high (SENS^{HIGH}; #16, #30) sensitivity to CHK1i (**e**) (*see* **Supplementary Fig. S1a** and **Materials and Methods**) exposed to MRE11i and/or RAD11i for 96h as illustrated. Results are means±SEM. Number of independent experiments: 11 (**d**), 5 (**e**, #1SENS and #19SENS) and 6 (**e**, SENS^{HIGH} and SENS^{MED}). Data for SENS^{HIGH} and SENS^{MED} CRC-SCs were pooled. $^{*}P<0.05$, $^{**}P<0.01$, $^{***}P<0.001$ (one-way ANOVA and Bonferroni or Dunnett T3 post-hoc test), as indicated.

(**f**) Analysis of immunofluorescence microscopy of the reported RS markers in neoR-CRC-SCs (#1neoR and #19neoR) left untreated or treated for 24h with MRE11i (#1neoR: 25μM; #19neoR: 20μM) and/or RAD51i (#1neoR: 7.5μM; #19neoR: 5μM) upon staining with an antibody recognizing γH2AX. Quantification of γH2AX^{+} cells (>5 foci) is reported. Results are means±SEM and individual data points of two independent experiments. No statistical differences between treated and untreated conditions were observed (Kruskal-Wallis test and Dunn's post-hoc test).

(**g**) NeoR-CRC-SCs (#19neoR) left untreated or administered with 20μM MRE11i and/or 5μM RAD51i were fixed, costained with DAPI and an antibody recognizing phospho-histone H2AX (γH2AX), and then subjected to flow cytometry analysis. Quantification of cells in G$_2$/M-phase or displaying DNA lesions (γH2AX^{+}) in S-phase or G$_2$/M-phase are reported. Results are means±SEM and individual data points of 3 independent experiments. *See* also **Fig. 7f**. $^{*}P<0.05$, $^{**}P<0.01$, $^{***}P<0.001$ (one-way ANOVA and Bonferroni post-hoc test), as indicated (left histogram). Statistical analysis for γH2AX^{+} S-phase or G$_2$/M-phase cells was not performed given the low percentage (≤3%) of positive cells for all conditions. All significant *P* values are shown in **Supplementary Table S4**.

Supplementary Figure S8. Related to Figure 7.

Induction of regulated cell death by MRE11 and RAD51 in CSCs.

(**a,b**) Live fluorescence microscopy in #19neoR-CRC-SCs left untreated or treated with 20µM MRE11i, and/or 7.5µM RAD51i in the presence or not of the caspase inhibitor Q-VD-Oph at 25µM (**a**) or RIPK3 inhibitor GSK'872 at 30µM (**b**) and then co-stained with the vital dye propidium iodide (PI) and the DNA Hoechst 33342 (to counterstain nuclei). Representative images from 1 out of 4 (**a**) or 2 (**b**) independent experiments showing PI incorporation into cells (a sign of regulated cell death induction) are reported. Note that caspase but not RIPK3 inhibition decreases PI incorporation due to MRE11i+RAD51i administration. Scale bar: 10µm. *See* **Materials and Methods** for further details.

References

1. Manic, G.; Signore, M.; Sistigu, A.; Russo, G.; Corradi, F.; Siteni, S., et al. CHK1-targeted therapy to deplete DNA replication-stressed, p53-deficient, hyperdiploid colorectal cancer stem cells. Gut 2018, 67, 903-917, doi:10.1136/gutjnl-2016-312623.
