# Supplementary figures and images for: Control of replication stress and mitosis in colorectal cancer stem cells through the interplay of PARP1, MRE11 and RAD51
Source: Cell Death Differ. 2021 Feb 2;28(7):2060–82. doi: 10.1038/s41418-020-00733-4 (PMC8257675; doi:10.1038/s41418-020-00733-4)

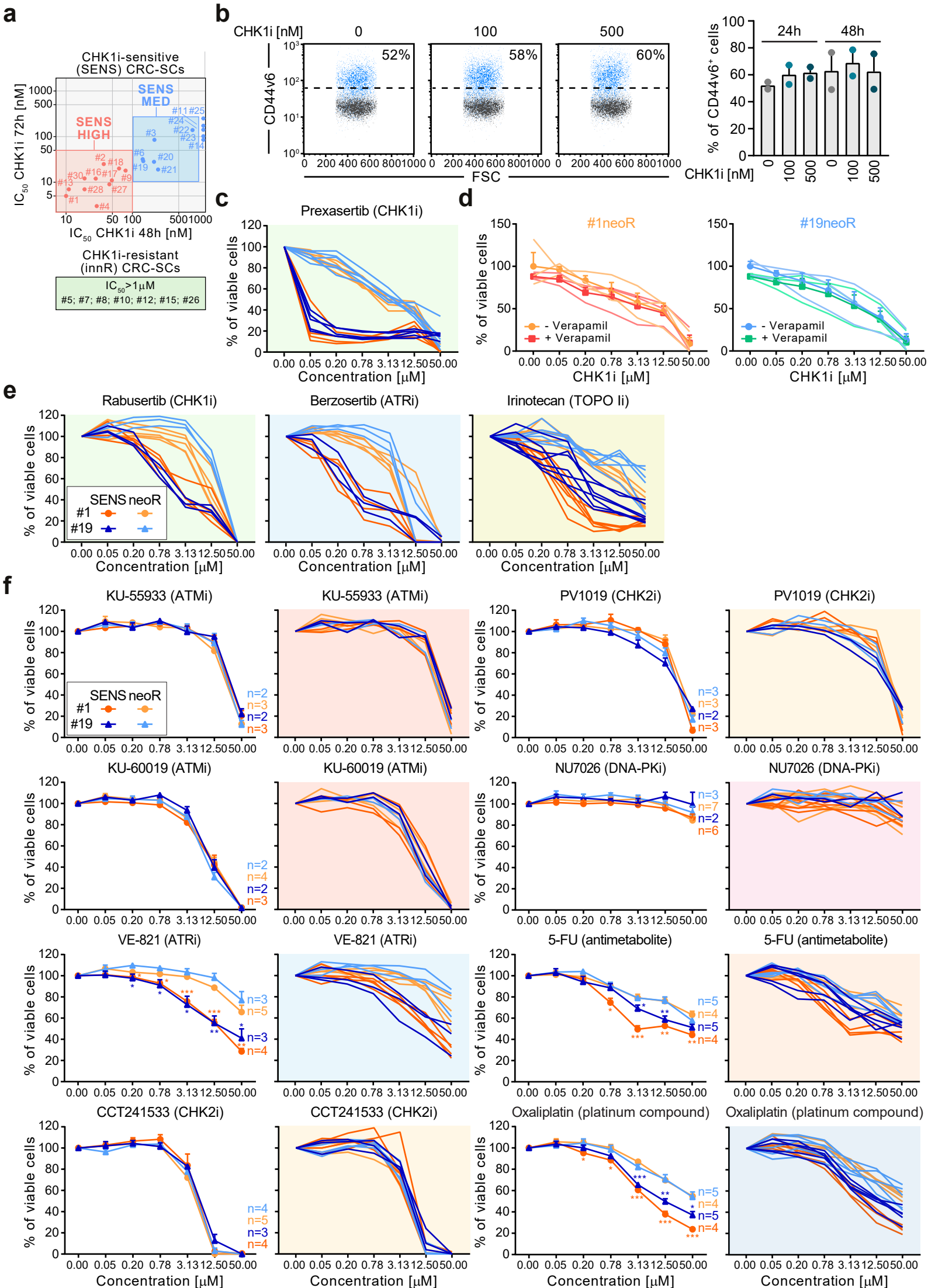

**FIGURE S1**

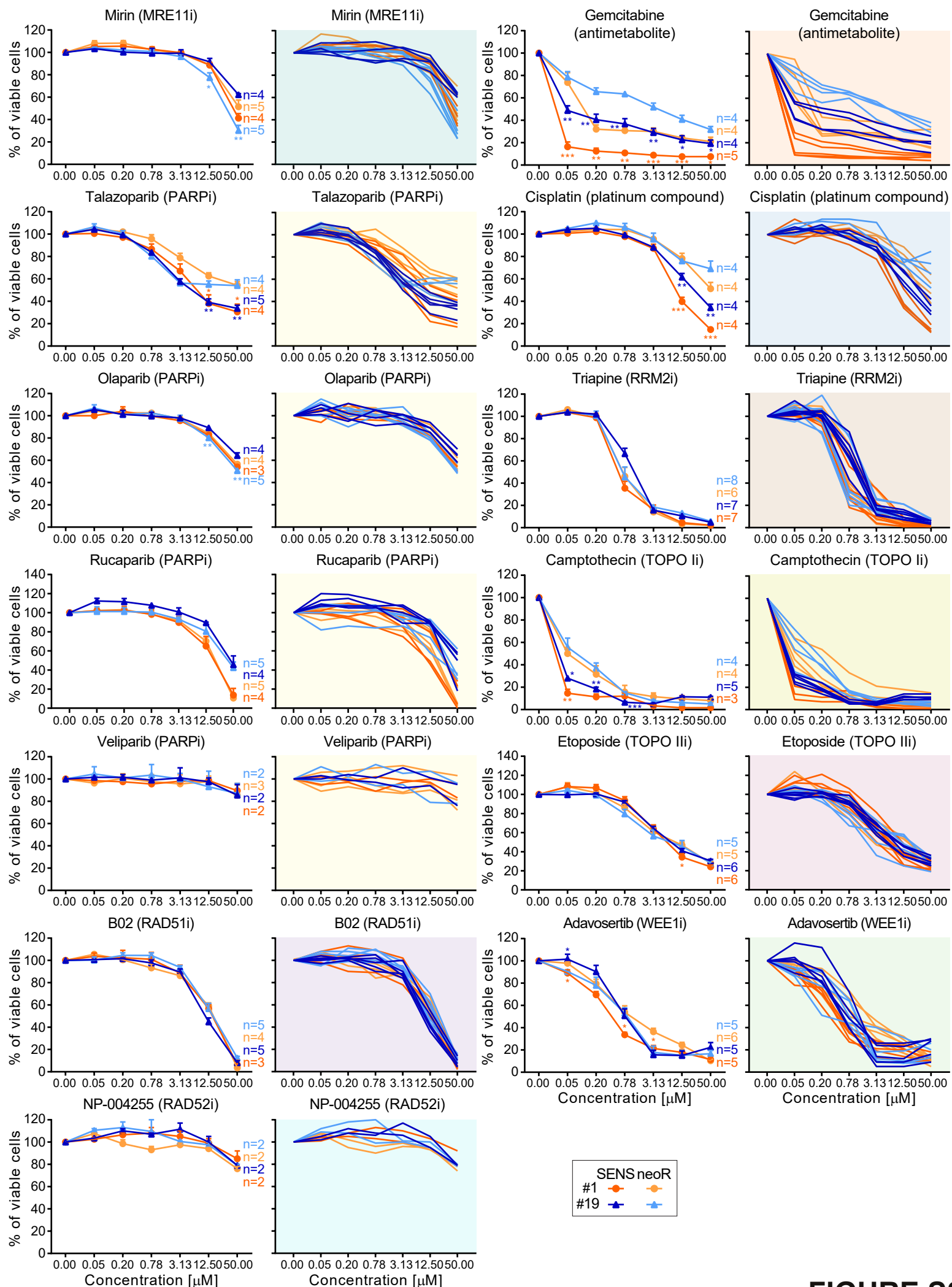

**FIGURE S2**

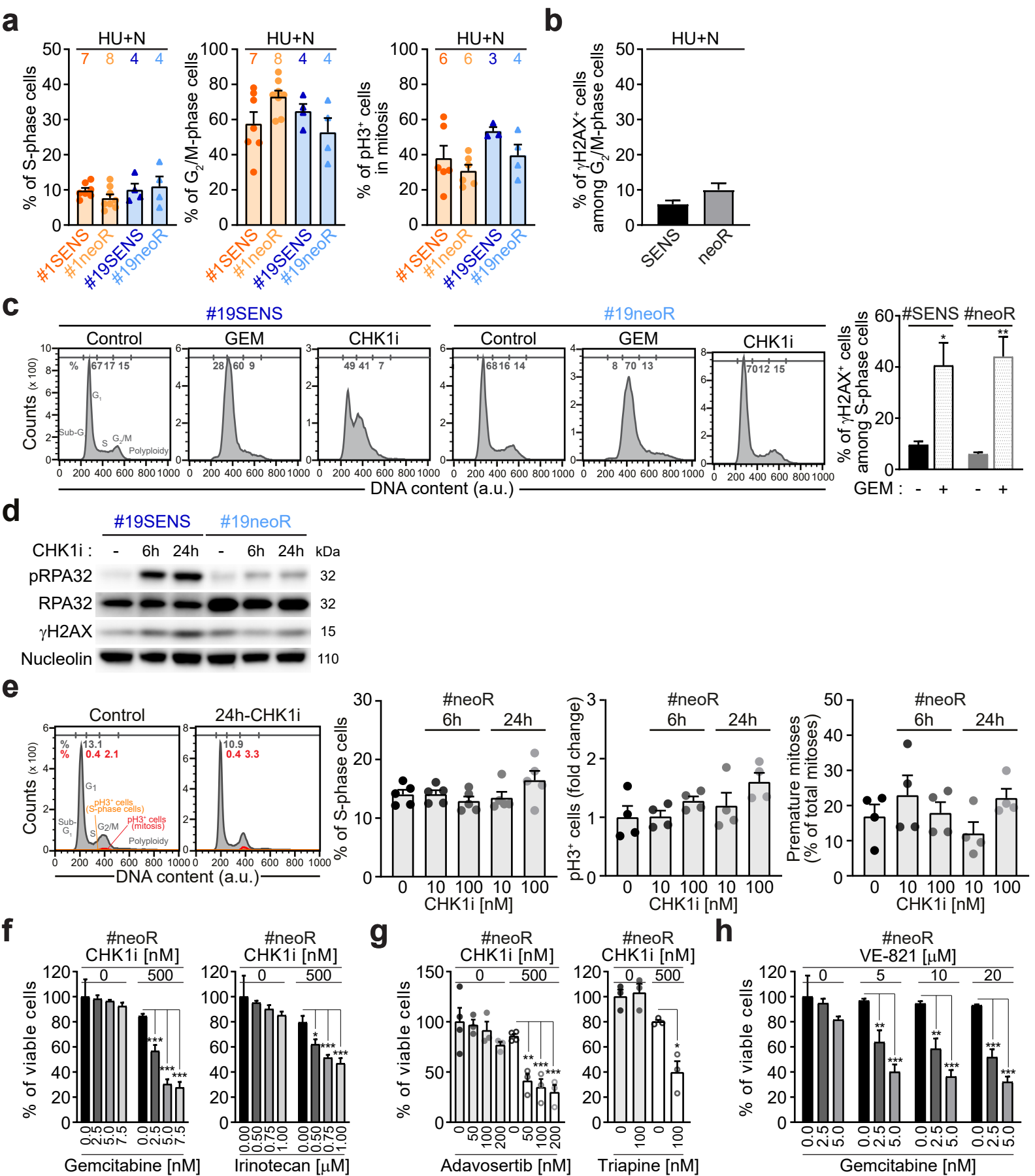

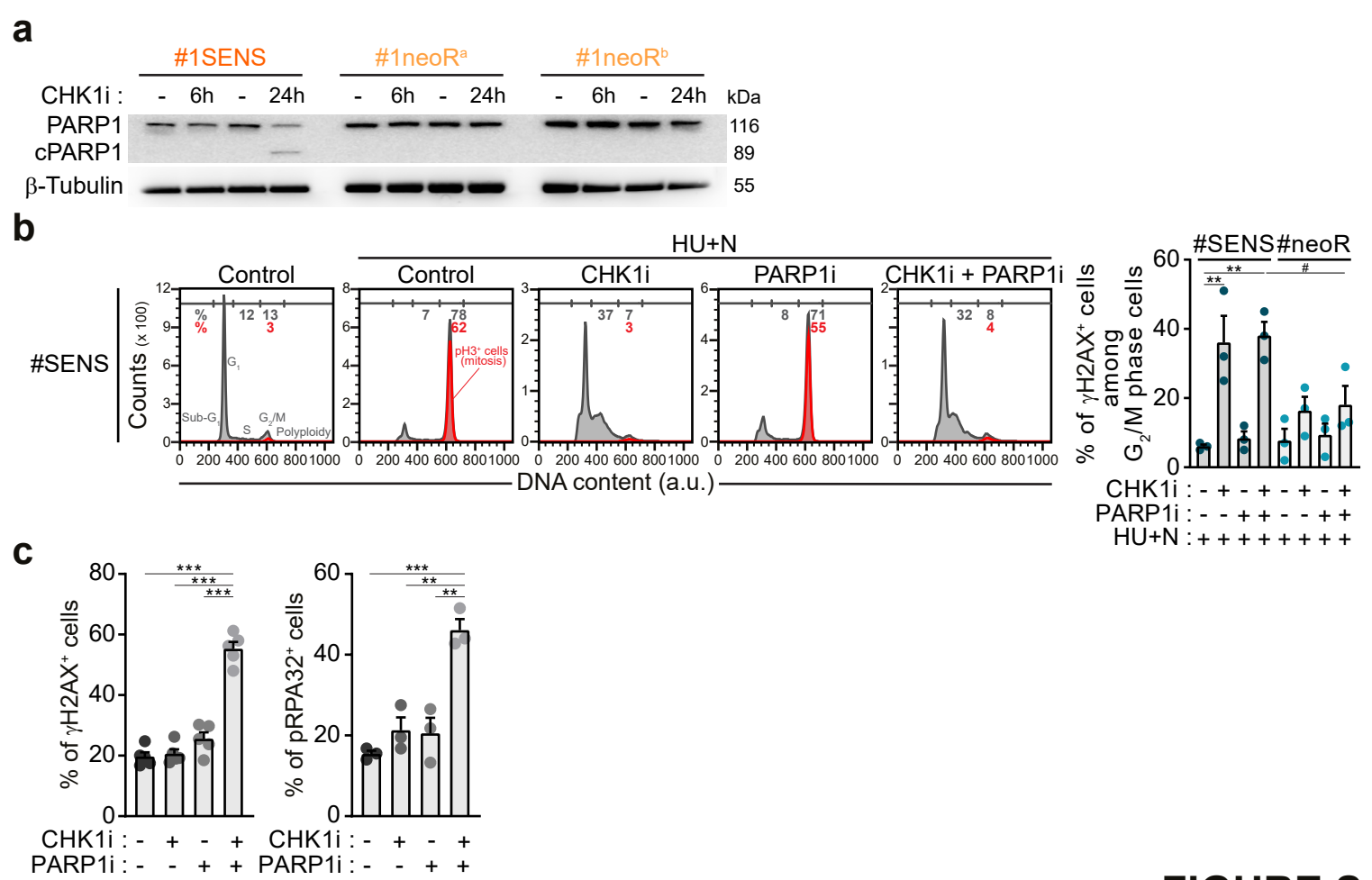

**FIGURE S4**

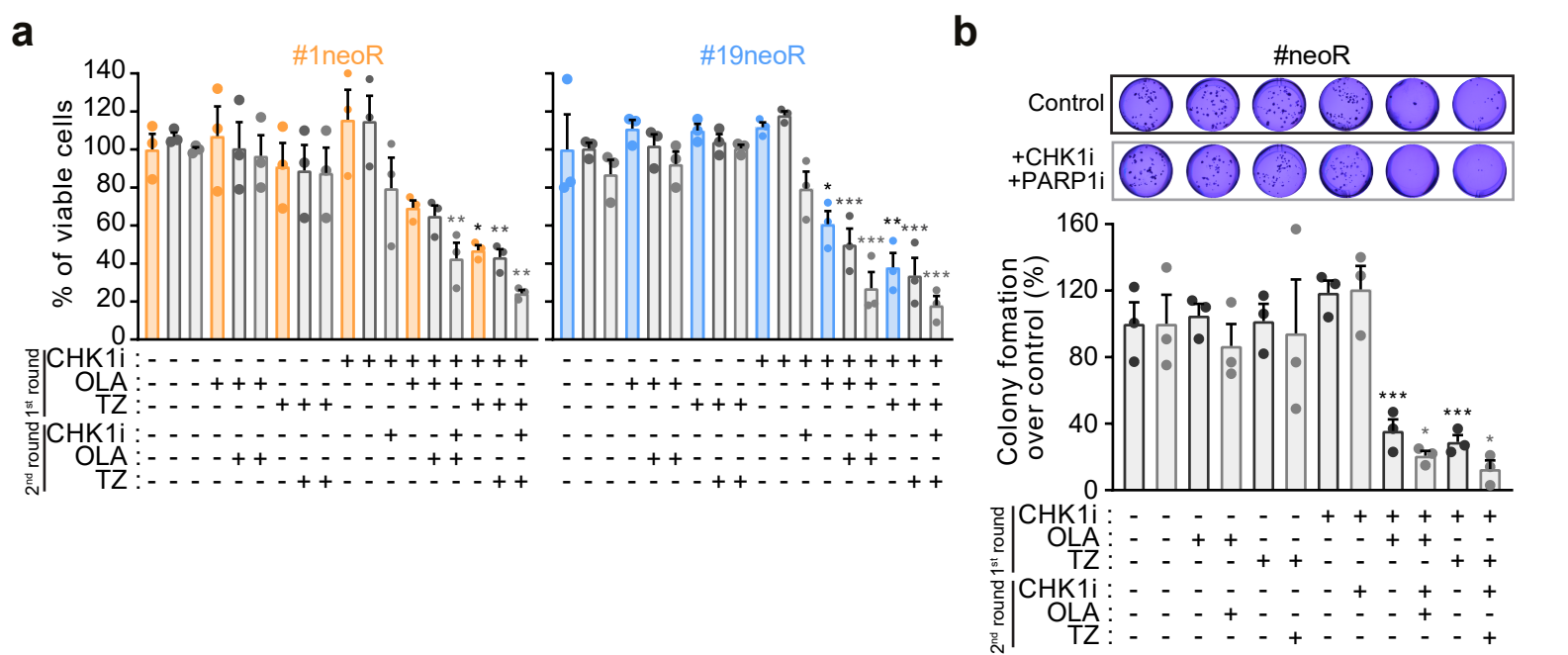

**FIGURE S5**

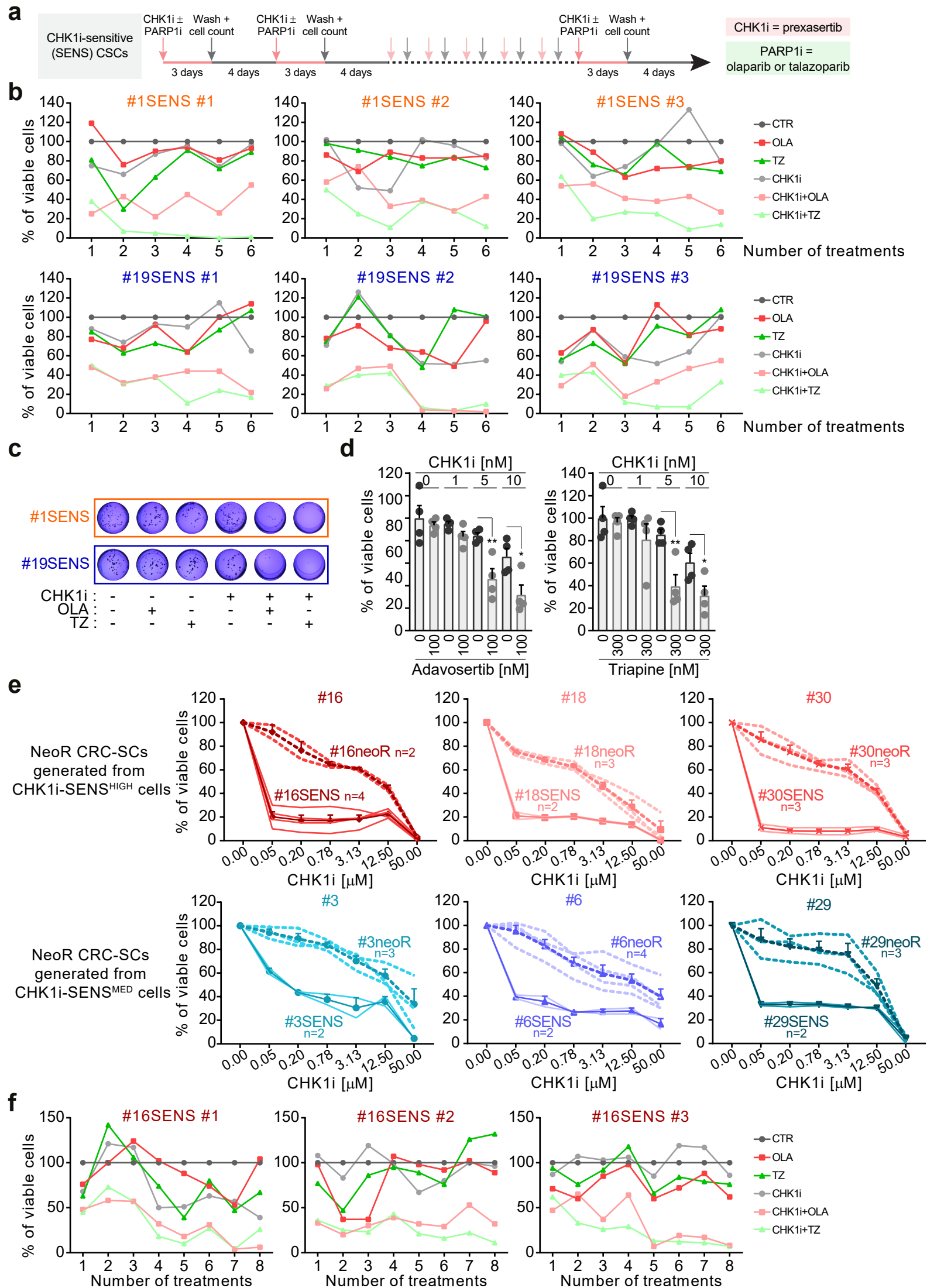

**FIGURE S6**

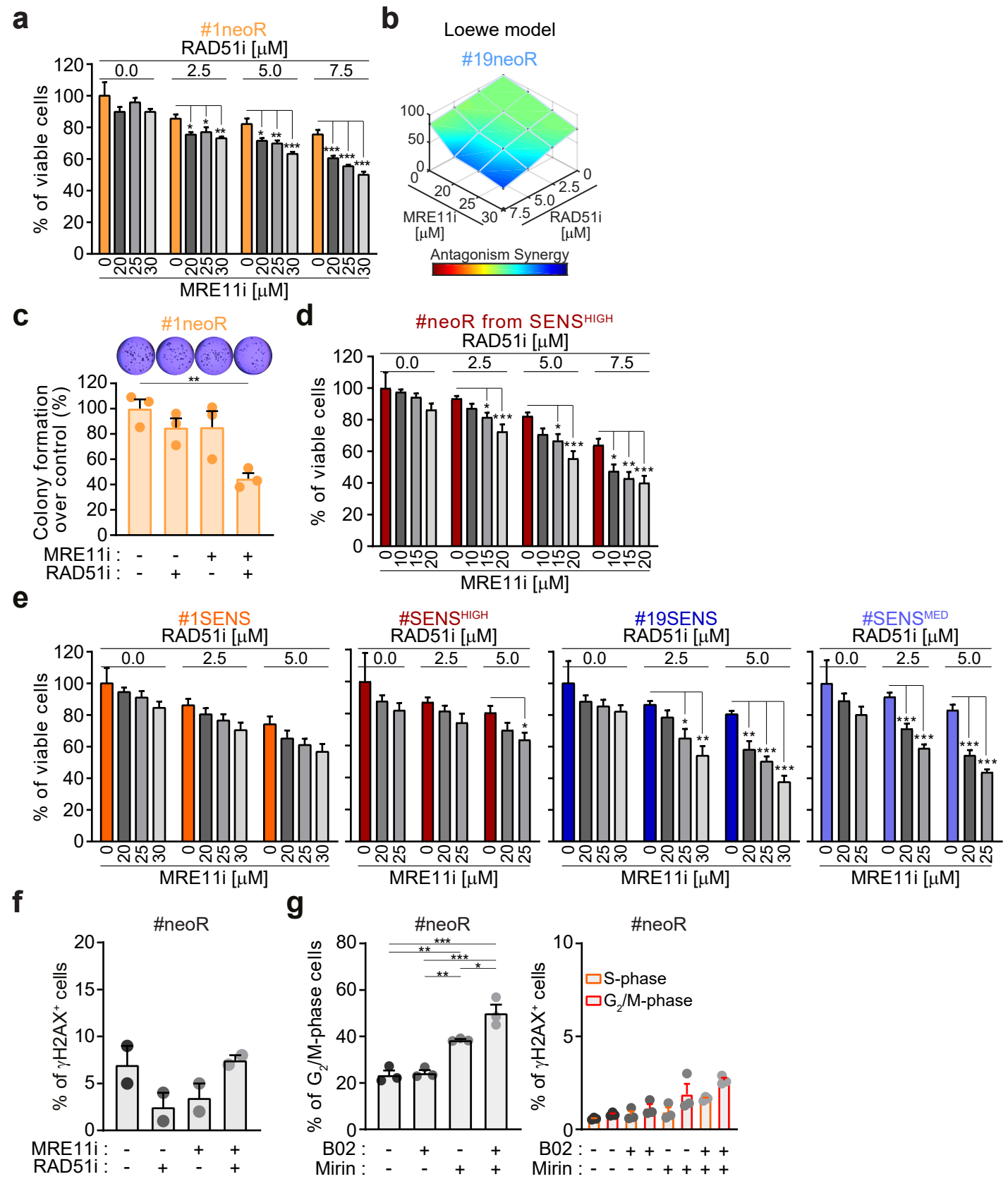

**FIGURE S7**

**a**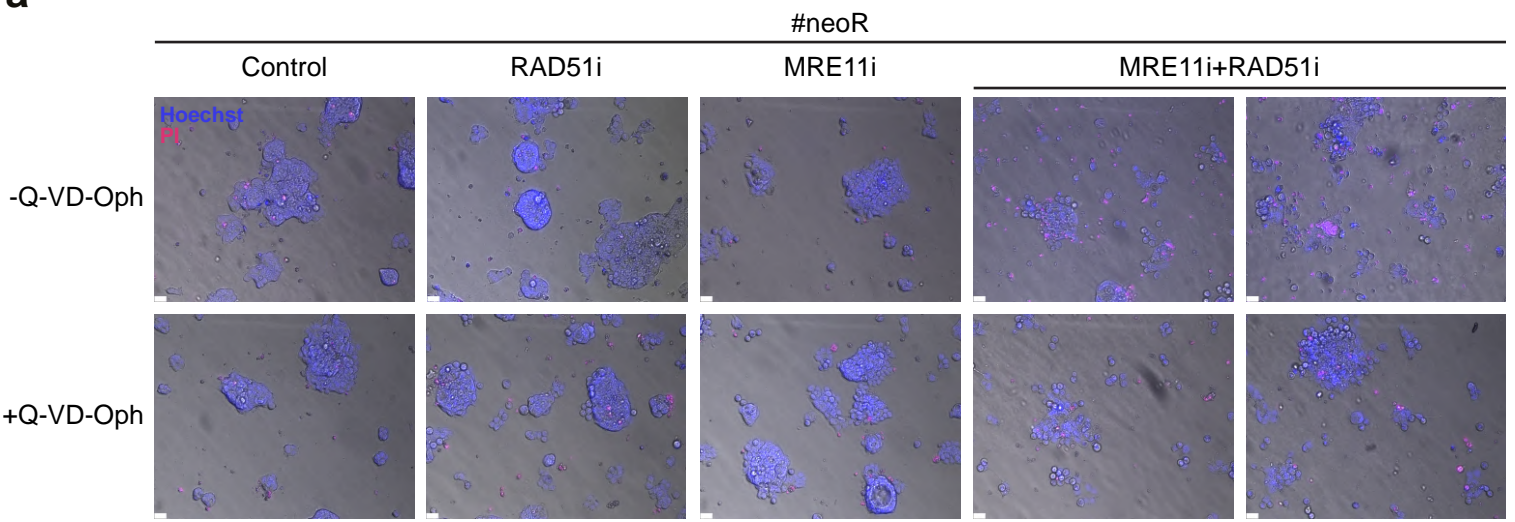**b**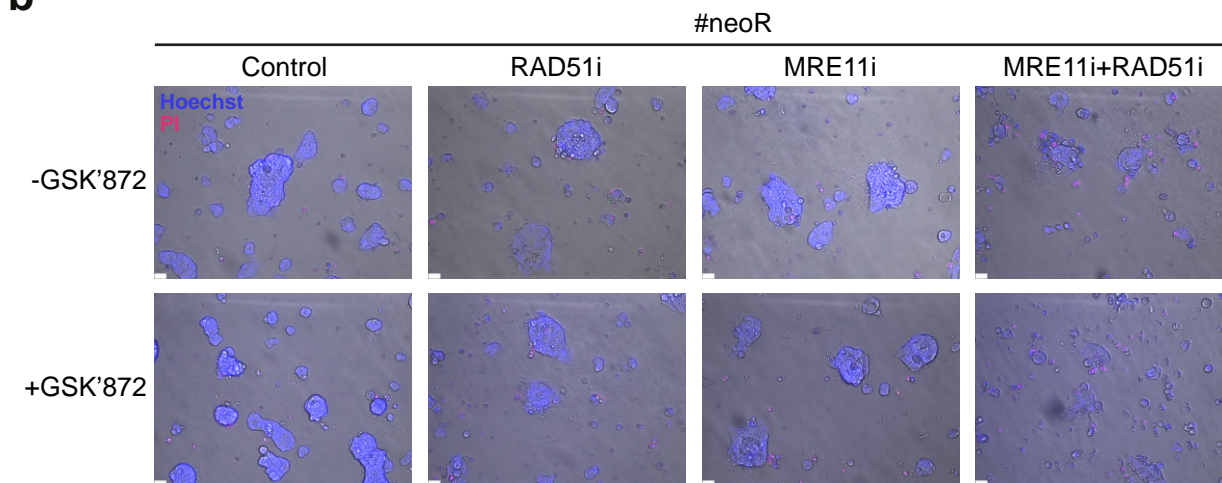**FIGURE S8**

Supplement: Supplementary file 2 — Supplementary Figures [file 41418_2020_733_MOESM2_ESM.pdf]
