# Supplementary material for: Control of replication stress and mitosis in colorectal cancer stem cells through the interplay of PARP1, MRE11 and RAD51
Source: Cell Death Differ. 2021 Feb 2;28(7):2060–82. doi: 10.1038/s41418-020-00733-4 (PMC8257675; doi:10.1038/s41418-020-00733-4)
Supplement: Supplementary file 3 — Supplementary Table S1_Manic et al_CDD-20-1279.RR [file 41418_2020_733_MOESM3_ESM.docx]

**TABLE S1: List of IC_50_ mean values^1^ for drugs reported in Fig. 1e. Related to Fig. 1 and S1.**

| Class of drug | Drug | CSCs | IC_50_ #SENS [nM] | IC_50_ #neoR [nM] |
| --- | --- | --- | --- | --- |
| ATM inhibitors | KU-55933 | #1 | 33212 | 23133 |
|  |  | #19 | 32998 | 26883 |
|  | KU-60019 | #1 | 9371 | 10595 |
|  |  | #19 | 10416 | 8360 |
| ATR inhibitors | VE-821 | #1 | 16108 | 49141 |
|  |  | #19 | 29666 | >50000 |
|  | Berzosertib | #1 | 548 | 6917 |
|  |  | #19 | 698 | 6441 |
| CHK1 inhibitors | Rabusertib | #1 | 3123 | 11551 |
|  |  | #19 | 2258 | 14647 |
|  | Prexasertib | #1 | <49 | 2861 |
|  |  | #19 | <49 | 4132 |
| CHK2 inhibitors | CCT241533 | #1 | 5319 | 3685 |
|  |  | #19 | 5813 | 4152 |
|  | PV1019 | #1 | 22476 | 34339 |
|  |  | #19 | 22864 | 23605 |
| DNA-PK inhibitor | NU7026 | #1 | >50000 | >50000 |
|  |  | #19 | >50000 | >50000 |
| MRE11 inhibitor | Mirin | #1 | 41118 | 44328 |
|  |  | #19 | >50000 | 30048 |
| PARP inhibitors | Talazoparib | #1 | 13004 | 41108 |
|  |  | #19 | 9077 | 33650 |
|  | Olaparib | #1 | >50000 | >50000 |
|  |  | #19 | >50000 | 48285 |
|  | Rucaparib | #1 | 19107 | 19171 |
|  |  | #19 | 44621 | 36936 |
|  | Veliparib | #1 | >50000 | >50000 |
|  |  | #19 | >50000 | >50000 |
| RAD51 inhibitor | B02 | #1 | 13558 | 13988 |
|  |  | #19 | 11239 | 14898 |
| RAD52 inhibitor | NP004255 | #1 | >50000 | >50000 |
|  |  | #19 | >50000 | >50000 |
| Anti-metabolites | 5-FU | #1 | 16314 | >50000 |
|  |  | #19 | 38740 | >50000 |
|  | Gemcitabine | #1 | <49 | 298 |
|  |  | #19 | 178 | 4414 |
| Platinum compounds | Cisplatin | #1 | 10439 | 42635 |
|  |  | #19 | 23933 | >50000 |
|  | Oxaliplatin | #1 | 7376 | 47706 |
|  |  | #19 | 16129 | 41931 |
| RRM2 inhibitor | Triapine | #1 | 803 | 906 |
|  |  | #19 | 1231 | 976 |
| TOPO I inhibitors | Camptothecin | #1 | <49 | 91 |
|  |  | #19 | <49 | 93 |
|  | Irinotecan | #1 | 789 | 24062 |
|  |  | #19 | 4513 | 39770 |
| TOPO II inhibitor | Etoposide | #1 | 7925 | 10455 |
|  |  | #19 | 10170 | 9721 |
| WEE1 inhibitor | Adavosertib | #1 | 534 | 1606 |
|  |  | #19 | 1056 | 861 |

^1^When non calculable, IC_50_ values have been arbitrarily set to 49 or 50000 nM, *i.e.*, the lowest and highest concentration of the dose-response. In this case, 49 and 50000 nM have been used to calculate the IC_50_ mean value.

TOPO I/II, topoisomerase I/II.
