# Supplementary material for: Control of replication stress and mitosis in colorectal cancer stem cells through the interplay of PARP1, MRE11 and RAD51
Source: Cell Death Differ. 2021 Feb 2;28(7):2060–82. doi: 10.1038/s41418-020-00733-4 (PMC8257675; doi:10.1038/s41418-020-00733-4)
Supplement: Supplementary file 4 — Supplementary Table S2_Manic et al_CDD-20-1279.RR [file 41418_2020_733_MOESM4_ESM.docx]

**TABLE S2: List of all log_2_ values reported in Figure 1e. Related to Figure 1 and S1.**

| Concentrations [µM] | 0.05 | 0.20 | 0.78 | 3.13 | 12.50 | 50.00 | 0.05 | 0.20 | 0.78 | 3.13 | 12.50 | 50.00 |
| --- | --- | --- | --- | --- | --- | --- | --- | --- | --- | --- | --- | --- |
| DRUG | Log_2_ #1neoR/#1SENS | | | | | | Log_2_ #19neoR/#19SENS | | | | | |
| KU-55933 | 0,08 | 0,05 | -0,05 | 0,00 | -0,13 | -0,71 | -0,03 | 0,01 | -0,01 | 0,04 | -0,09 | -0,87 |
| KU-60019 | 0,07 | 0,05 | 0,07 | 0,07 | 0,08 | 0,26 | -0,02 | 0,01 | -0,07 | -0,10 | -0,37 | 0,42 |
| VE-821 | 0,08 | 0,06 | 0,13 | 0,39 | 0,68 | 1,20 | 0,08 | 0,17 | 0,24 | 0,51 | 0,83 | 0,91 |
| Berzosertib | 0,25 | 0,65 | 1,37 | 1,28 | 6,60 | 5,32 | 0,17 | 0,60 | 1,54 | 1,77 | -0,35 | -0,26 |
| Rabusertib | 0,05 | 0,13 | 0,48 | 1,06 | 0,62 | 2,00 | 0,00 | 0,24 | 0,79 | 1,59 | 1,36 | 0,00 |
| Prexasertib | 1,98 | 2,65 | 2,50 | 1,70 | 0,93 | 1,42 | 1,62 | 2,20 | 2,18 | 1,79 | 1,10 | -0,28 |
| CCT241533 | -0,01 | -0,03 | -0,09 | -0,21 | -3,64 | 1,00 | -0,08 | -0,02 | 0,03 | -0,09 | -2,08 | 0,00 |
| PV1019 | -0,05 | -0,02 | -0,11 | 0,00 | 0,06 | 1,72 | -0,03 | 0,09 | 0,09 | 0,14 | 0,19 | -0,67 |
| NU7026 | 0,04 | 0,03 | 0,03 | 0,08 | 0,04 | -0,04 | 0,03 | 0,01 | 0,07 | 0,06 | -0,09 | -0,11 |
| Mirin | 0,04 | 0,04 | -0,01 | -0,01 | 0,00 | 0,32 | 0,01 | 0,02 | 0,02 | -0,05 | -0,24 | -1,04 |
| Talazoparib | 0,06 | 0,07 | 0,15 | 0,23 | 0,72 | 0,83 | 0,03 | 0,02 | -0,06 | -0,03 | 0,50 | 0,68 |
| Olaparib | 0,06 | -0,02 | 0,04 | 0,02 | 0,03 | 0,07 | 0,02 | 0,00 | 0,04 | -0,01 | -0,15 | -0,34 |
| Rucaparib | 0,00 | 0,01 | -0,01 | 0,03 | 0,11 | -0,38 | -0,15 | -0,14 | -0,10 | -0,11 | -0,15 | -0,10 |
| Veliparib | -0,03 | 0,05 | 0,03 | -0,03 | -0,01 | -0,07 | 0,04 | -0,01 | 0,06 | -0,01 | -0,07 | 0,03 |
| B02 | 0,03 | -0,03 | -0,12 | -0,04 | 0,06 | -0,86 | -0,01 | 0,04 | 0,09 | 0,07 | 0,34 | 0,63 |
| NP-004255 | 0,07 | -0,11 | -0,22 | -0,11 | -0,07 | -0,16 | 0,09 | 0,04 | 0,03 | -0,15 | -0,04 | 0,00 |
| 5-FU | -0,02 | 0,03 | 0,27 | 0,68 | 0,53 | 0,52 | 0,01 | 0,14 | 0,04 | 0,20 | 0,38 | 0,17 |
| Gemcitabine | 2,17 | 1,37 | 1,51 | 1,77 | 1,67 | 1,47 | 0,69 | 0,69 | 0,79 | 0,84 | 0,86 | 0,72 |
| Cisplatin | 0,02 | 0,03 | 0,09 | 0,13 | 0,98 | 1,80 | 0,01 | 0,06 | 0,09 | 0,11 | 0,31 | 1,01 |
| Oxaliplatin | 0,01 | 0,14 | 0,18 | 0,52 | 0,89 | 1,20 | -0,02 | 0,06 | 0,09 | 0,33 | 0,50 | 0,55 |
| Triapine | 0,04 | 0,02 | 0,38 | -0,22 | -0,46 | 0,02 | 0,01 | -0,02 | -0,55 | 0,24 | 0,31 | 0,24 |
| Camptothecin | 1,77 | 1,47 | 0,39 | 1,79 | 2,51 | 2,26 | 0,99 | 1,01 | 1,18 | 0,58 | -0,87 | -1,14 |
| Irinotecan | 0,05 | 0,40 | 1,16 | 2,03 | 2,07 | 0,94 | 0,06 | 0,34 | 0,42 | 0,79 | 1,14 | 1,01 |
| Etoposide | 0,00 | -0,07 | -0,12 | -0,07 | 0,45 | 0,21 | 0,06 | -0,02 | -0,21 | -0,19 | 0,16 | -0,11 |
| Adavosertib | 0,14 | 0,19 | 0,68 | 0,76 | 0,45 | -0,14 | -0,17 | -0,21 | 0,06 | 0,19 | 0,02 | -0,43 |
